# Nutrition Recommendations for People Living After Cancer: A Systematic Review and Critical Appraisal of Clinical Practice Guidelines

**DOI:** 10.3390/nu18101516

**Published:** 2026-05-09

**Authors:** Lauren Ducat, Ekavi N. Georgousopoulou, Nathan M. D’Cunha, Murray R. Turner, Jane Kellett

**Affiliations:** 1 Discipline of Nutrition and Dietetics, Faculty of Health, University of Canberra, Canberra, ACT 2617, Australia; lauren.ducat@canberra.edu.au (L.D.); ekavi.georgousopoulou@canberra.edu.au (E.N.G.); nathan.dcunha@canberra.edu.au (N.M.D.); 2 Centre for Ageing Research and Translation, University of Canberra, Canberra, ACT 2617, Australia; 3 Faculty of Health, University of Canberra, Canberra, ACT 2617, Australia; murray.turner@canberra.edu.au

**Keywords:** systematic review, practice guideline, quality assessment, cancer survivor, nutrition, breast cancer, colorectal cancer, prostate cancer, head and neck cancer

## Abstract

**Background/Objectives**: Clinical practice guidelines are essential tools for ensuring evidence-based, standardised care for people living after cancer; however, the quality of international clinical practice guidelines with nutrition recommendations for this population has not been assessed. This systematic review aimed to critically appraise the quality of international clinical practice guidelines for the nutritional care of people living after cancer and to summarise common recommendations for both the overall condition and for breast, colorectal, prostate, and head and neck cancers. **Methods**: Guidelines providing recommendations for nutritional management in people living after cancer were comprehensively searched in five electronic databases (PubMed, Web of Science Core Collection, CINAHL, SCOPUS, and Google Search). Included guidelines were appraised using the AGREE II and AGREE-REX tools. **Results**: Twenty-two clinical practice guidelines met the inclusion criteria. From the AGREE tools, ten guidelines were rated as “recommended”, eight guidelines as “recommended with modifications”, and four guidelines as “not recommended”. The AGREE II domains with the highest mean percentages were Editorial Independence (81%, range = 46–100%), Clarity of Presentation (80%, range = 58–94%), and Scope and Purpose (79%, range = 42–100%); the domain with the lowest mean percentage was Applicability (39%, range = 6–77%). The AGREE-REX item with the highest mean percentage was Evidence (72%, range = 25–100%), while the items with the lowest mean percentage were Values and Preferences of Patients/Populations (25%, range = 0–75%) and Values and Preferences of Policy/Decision-Makers (25%, range = 0–83%). The most common nutrition recommendations provided were weight management; increased consumption of fruits, vegetables, and wholegrains; strategies for symptom management; and limitation of specific carcinogenic foods. Twelve clinical practice guidelines provided specific recommendations for living after different cancer types, with the most commonly considered types being head and neck cancer, breast cancer, colorectal cancer, and prostate cancer. **Conclusions**: While many guidelines were rated highly for clarity and Editorial Independence, most were limited in practical applicability and in their consideration of patient and policymaker values. The findings underline the necessity for more robust, comprehensive, and patient-centred guideline development to support consistent, evidence-based nutritional recommendations for people living after cancer worldwide.

## 1. Introduction

Clinical practice guidelines are commonly used by health professionals, policy developers, and patients to guide evidence-based decision-making [1]. These guidelines are designed to ensure that the quality of care is maintained, care decisions are standardised, and new and emerging evidence is translated into clinical practice [2]. However, the quality of these guidelines varies widely, with unclear recommendations, results potentially biassed by panel selection, and infrequent revision schedules all impacting on their effectiveness [2]. This can lead to sub-optimal care and inefficient use of resources [3].

Cancer is one of the largest health priorities for research and treatment internationally due to the burden the disease places on people with cancer, their families, and healthcare systems [4]. As treatment options continue to improve, the number of people living after cancer also continues to grow, with approximately 53.5 million people living around the world who have been diagnosed with cancer in the past five years, and the number of people living for ten or more years after diagnosis is rising [5,6,7,8]. Life beyond cancer is an important stage in the clinical treatment pathway, with many people living after cancer experiencing late and long-term effects both physically and psychologically [9]. Symptoms including fatigue, trouble sleeping, digestive issues, urinary issues, changes in focus, and changes in sight, smell, and hearing are common across different cancer types and treatment modalities and can greatly impact quality of life [9,10]. Nutrition-related issues like weight loss, malnutrition, weight gain, alcoholism, and sarcopenia also disproportionately affect different groups living after cancer, with susceptibility depending on the location and severity of malignancy, and the methods of cancer treatment employed [11]. People living after cancer are also more vulnerable to several chronic diseases, including heart disease, diabetes, chronic obstructive pulmonary disorder, liver disease, kidney disease, stroke and hypertension [12].

Given the complexity of the vulnerabilities and concerns of people living after cancer, research continues to emerge around appropriate screening, assessment, and treatment for this group. Nutrition clinical practice guidelines have been published since at least 2001 to assist clinicians working with people living after cancer [13]. Research published in 2024 assessed the quality of clinical practice guidelines for people living after cancer by comparing them with the Dietary Guidelines for Americans; however, this research has an established American focus, considering only five guidelines published in the United States [14]. Moreover, no research to date has systematically assessed international clinical practice guidelines. Different continents and countries diverge in cancer incidence and mortality; in availability and accessibility of cancer care; in food system policies, priorities, and healthy eating guidelines; in nutritional deficiencies and health inequality; and in sociocultural influences on food choice [6,15,16,17,18,19]. Because of these dissimilarities, research that considers only the availability of American research and analyses it only in the context of American healthy eating guidelines has limited external validity in a global context, reducing the applicability of these results for clinicians and researchers outside the United States.

To establish the quality of guidelines and the strength and limitations of their recommendations, the Appraisal of Guidelines for Research and Evaluation (AGREE) Trust have developed several ratified tools. The Appraisal of Guidelines for Research and Evaluation: second edition (AGREE II) framework and the Appraisal of Guidelines for Research and Evaluation: Recommendation EXcellence (AGREE-REX) tools are designed to assess the quality of clinical practice guidelines in terms of methodological rigour, clinical credibility, and implementability [20,21]. These tools have been used effectively in conjunction with one another by several publications to assess a range of different clinical practice guidelines [22,23].

This research aims to assess the quality of international clinical practice guidelines for people living after cancer, using the validated tools AGREE II and AGREE-REX to provide evidence and guidance for healthcare providers and policymakers seeking to use these guidelines to shape their practice. The research also aims to identify and describe key themes in nutritional recommendations provided by these clinical practice guidelines, both overall and by major cancer subtype, to facilitate comparison and clarify decision-making for clinicians.

## 2. Materials and Methods

A critical and systematic appraisal of the quality of clinical practice guidelines for the nutritional care of people living after cancer was conducted using the AGREE II and AGREE-REX instruments. The protocol was registered a priori with PROSPERO (CRD420250634405) on 7 April 2025.

### 2.1. Data Sources and Searches

A systematic literature review search for English-language articles in PubMed (U.S. National Library of Medicine), Web of Science Core Collection (Clarivate), CINAHL (EBSCO), and SCOPUS (Elsevier) was initially conducted on 21 April 2025 and last updated on 21 April 2026 with no restriction on date of publication. A Boolean search strategy was employed, with slight translations according to database:

(guideline* OR recommendation* OR statement*) AND (nutrition* OR diet* OR eating) AND (cancer survivor* OR “after cancer” OR “survivors of cancer” OR “post-cancer”).

To source the grey literature, the Google™ engine Web Search was used to source non-journal records that may not have been listed on the above bibliographic databases, with all 119 results considered.

### 2.2. Inclusion and Exclusion Criteria

Guidelines were included after assessment with the following inclusion and exclusion criteria:

Inclusion criteria: (a) full text available online, published in English or with English translation; (b) adults living after cancer or any subgroup of this group; (c) recommendations that constitute clinical practice guidelines; (d) guidelines published in any year; and (e) guidelines of any assessed quality.

Exclusion criteria: (a) consideration of only people with cancer rather than after cancer, or of people that have never had cancer; (b) guidelines that only consider minors (people under the age of 18 years); (c) publications or recommendations that do not constitute clinical practice guidelines (e.g., blog posts, news articles, and patient-directed information); (d) guidelines or recommendations provided by people or organisations without professional credentials or oversight; and (e) clinical practice guidelines that do not provide specificity on nutrition recommendations (e.g., general phrasing to “eat a healthy diet”).

### 2.3. Literature Screening and Data Extraction

**Figure 1:**
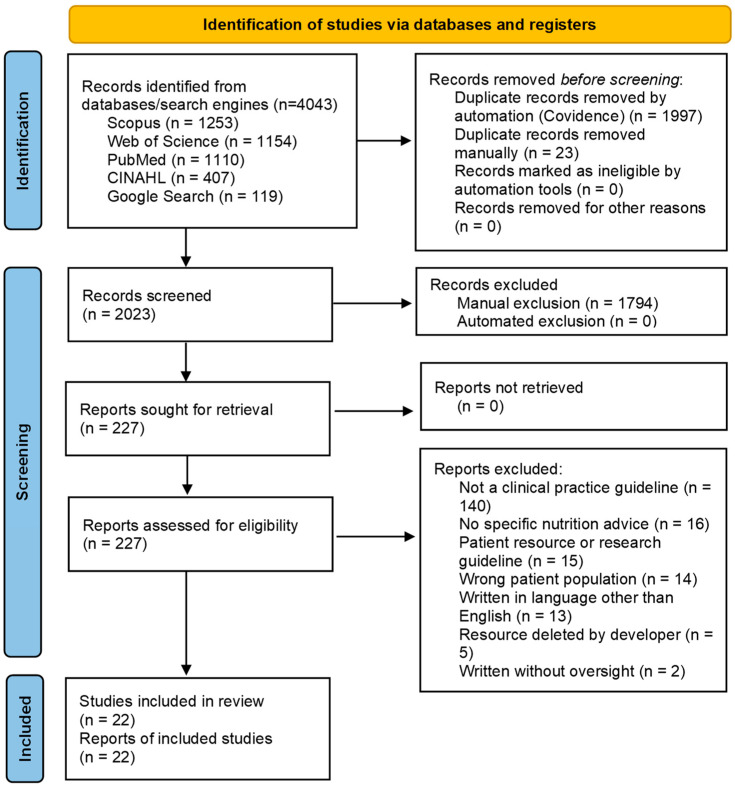
PRISMA flow diagram describing the selection and exclusion of studies.

Duplicates were identified and removed using Covidence software and during manual identification [24]. One reviewer reviewed all articles (L.D.), and two reviewers shared the role of independent second reviewer (J.K. and N.M.D.) to review all articles against the inclusion and exclusion criteria using Covidence, with another reviewer acting as moderator when needed (E.G.), first screening by title and abstract, and then by full text. To limit rater drift and variation in the individual judgement about the inclusion or exclusion of reports, several meeting sessions with all reviewers were dedicated to reviewer calibration and alignment of decision-making with the search protocol. The PRISMA 2020 flow diagram is available below (Figure 1) [25].

Data extraction was completed on included guidelines by the primary reviewer (L.D.) and reviewed by a second reviewer (role shared among J.K., E.G., and N.M.D.), including guideline developer, authors, year of publication, title, countries associated with development, populations considered, specific cancer sites considered, and sources of information.

For nutrition-specific recommendations, common categories of recommendations provided by the guideline were then extracted, both for the whole list of included guidelines and specified by cancer site (if included).

### 2.4. Quality Appraisal

The overall quality of each guideline for its intended purpose was assessed using the AGREE II and AGREE-REX instruments by the primary reviewer (L.D.) and independently by a second reviewer (role shared among J.K., E.G., and N.M.D). Prior to and at several points during the quality analysis process, all raters were engaged in meetings to ensure comprehensive understanding of the instruments and rater calibration.

The AGREE II instrument has 23 items and six domains. The six domains are Scope and Purpose, Stakeholder Involvement, Rigour Of Development, Clarity of Presentation, Applicability, and Editorial Independence. This instrument uses a Likert Scale, with each item being rated between 1 (Strongly Disagree) and 7 (Strongly Agree). Items that were not answerable, as the guideline did not provide any relevant information (or links to relevant information), were automatically rated a score of 1.

The AGREE-REX instrument has nine items and two overall evaluation items. The nine items are Evidence, Applicability to Target Users, Applicability to Patients/Populations, Values and Preferences of Target Users, Values and Preferences of Patients/Populations, Values and Preferences of Policy/Decision-Makers, Values and Preferences of Guideline Developers, Purpose, and Local Application and Adoption. The two overall evaluation items are “I would recommend these guideline recommendations for use in the appropriate context”, and “I would recommend these guideline recommendations for use in my context”. These overall evaluation items are subjective, based on the clinical judgement of the reviewers after completion of the quality appraisal, and are not directly derived from the score the guideline has achieved on either AGREE II tool. The second evaluation item (“-in my context”) is optional and has been omitted because it is not relevant to this review. The AGREE-REX instrument also ranks each of the nine items on a Likert Scale from 1 (Lowest Quality) to 7 (Highest Quality). The overall evaluation item can be marked with any of three options: “yes”, “yes, with modifications”, or “no”.

The score for each item or domain was compiled, then the results were translated to scaled scores using the following formula, as recommended by the AGREE instrument:

$$Scaled\;Score=\;\frac{Obtained\;Score-Minimum\;Possible\;Score}{Maximum\;Possible\;Score-Minimum\;Possible\;Score}\times 100\%$$

The scaled score for each item or domain ranges from a possible 0% to 100%. A higher percentage in each domain or item indicates that a guideline is of higher quality for this section. The AGREE II and AGREE-REX instruments do not provide any recommendations or thresholds for guideline quality or for whether guidelines should be recommended, with the results designed for comparison between guidelines and domains. Determinations of which guidelines are suitable for use under which circumstances should be determined using the clinical judgement of the healthcare provider and the priorities of the consumer.

Inter-rater reliability for quality appraisal tools was assessed using the intraclass correlation coefficient (ICC) based on a two-way mixed-effects, absolute-agreement, average-rating model of scaled scores determined using SPSS Statistics software (Version 31.0; IBM Corp., 2025, Armonk, NY, USA). The average measures and 95% CI have been reported.

## 3. Results

### 3.1. Study Selection

From a record search conducted on 21 April 2026, 22 clinical practice guidelines met eligibility criteria. Full details of study selection are available in Figure 1.

### 3.2. Study Characteristics

Twenty-two clinical practice guideline articles met the inclusion criteria, as described in Table 1. Twenty-one publishers were associated with the development of these publications, with three of these papers created through collaboration between multiple organisations: a 2016 publication by the American Cancer Society (ACS) and the American Society for Clinical Oncology (ASCO), a 2020 publication by the World Cancer Research Fund (WCRF) and the American Institute of Cancer Research (AICR), and a 2021 publication written by twelve French organisations in intergroup collaboration (SNFGE, FFCD, GERCOR, UNICANCER, SFCD, SFED, SFRO, ACHBT, AFC, SFP-APA, SFNCM, and AFSOS) [26,27,28]. The American Cancer Society had the most articles included, with nine guidelines (one in collaboration with ASCO) published between 2001 and 2022 [13,27,29,30,31,32,33,34,35]. Other organisations that have published multiple guidelines that met inclusion criteria include the American Society of Clinical Oncology (three studies, one in collaboration with ACS) [27,36,37], ESPEN (two studies) [38,39], the National Comprehensive Cancer Network (NCCN, three studies) [40,41,42], and the WCRF (two studies, one in collaboration with the AICR) [28,43]. Considering the location of origin, 16 resources were produced by developers associated with the United States of America [13,27,28,29,30,31,32,33,34,35,36,37,40,41,42,44]; three resources originate from developers associated with Europe (one resource in conjunction with Australian researchers) [38,39,45]; one resource each originates from developers associated with Spain and France [26,46]; and one resource originates from an unspecified “international” team [43]. Most guidelines in this area were published from 2012 onwards, with the American Cancer Society being the only organisation to publish clinical practice guidelines before this point, with three guidelines published in 2001, 2003, and 2006 [13,29,30].

Most clinical practice guidelines report development using evidence from a literature review (n = 5), a systematic review (n = 5), expert consensus (n = 11), or a combination of these (n = 3) (Table 1). Six articles included evidence grading, with one article (a 2017 ESPEN publication) specifying the use of the Grading of Recommendations Assessment, Development, and Evaluation method [38], while the others did not specify a grading framework. Two studies drew on recommendations from other sources, with the 2015 publication from the American Society for Clinical Oncology summarising the organisation’s prior work [36]. The 2022 publication from the European Head and Neck Society adopted and adjusted recommendations from the American Cancer Society (2016 publication, also listed as an included guideline) [33,45].

Eleven of the 22 articles provided guidelines only for people living after cancer of unspecified type, with the other eleven providing some guidance on nutrition for people living after specific cancer types (Table 1). The most common cancer types that are considered with specificity are head and neck cancers (n = 6 guidelines) [13,29,30,33,34,45], breast cancer (n = 6 guidelines) [13,27,29,30,34,43], colorectal cancer (n = 5 guidelines) [13,29,30,32,34], and prostate cancer (n = 5 guidelines) [13,29,30,31,34]. Other cancer types referenced in the clinical practice guidelines include lung cancer, gastric cancer, gynaecological cancers, haematologic cancers, and genitourinary cancers.

Six clinical practice guidelines only provided recommendations for this review’s target population of people living after cancer [27,31,32,33,40,45]. The other 16 articles considered additional populations, including people with cancer and cancer patients (n = 11 guidelines) [26,28,35,36,38,39,41,42,43,44,46]; people with advanced cancer (n = 4 guidelines) [13,30,39,46]; people undergoing cancer treatment (n = 3 guidelines) [13,29,30]; people at risk of cancer (n = 2 guidelines) [28,36]; people in recovery from cancer treatment (n = 1 guideline) [30]; people with stable disease (n = 1 guideline) [34]; and people with nonmetastatic cancer (n = 1 guideline) [37]. The recommendations for populations that are not people living after cancer are not considered or reported in this review.

### 3.3. Quality Appraisal of Clinical Practice Guidelines Using the AGREE Tools

Considering the robustness of the quality appraisal process using the AGREE II instrument and AGREE-REX supplement, inter-rater reliability was determined to be high despite the methodology of this review including multiple reviewers sharing the same role: the average ICC measured from overall scaled scores was 0.83 (95% CI = 0.69–0.91), indicating good reliability. For the AGREE II instrument specifically, the average ICC measured from overall scaled scores was 0.82 (95% CI = 0.57–0.92). For the AGREE-REX supplement, the average ICC measured from overall scaled scores was 0.72 (95% CI = 0.34–0.88). All of these values show a good level of inter-rater reliability.

#### 3.3.1. AGREE II Instrument

Based on the AGREE II assessment results, the overall mean scaled score was 63% (range: 36–86%) (Table 2). The highest overall score was achieved by the 2016 publication by the ACS and ASCO, titled “American Cancer Society/American Society for Clinical Oncology Breast Cancer Survivorship Care Guideline”, with a scaled score of 86% (range = 50–100%) [27]. The 2017 guidelines by ESPEN, the 2019 publication by ASCO, and the 2022 guidelines by ACS on nutrition and physical activity all also scored highly, achieving 85%, 82%, and 82%, respectively [34,37,38]. The lowest overall rating was scored by the 2023 American Academy of Family Physicians guideline titled “Care of Cancer Survivors: Nutrition and Physical Activity”, with a scaled percentage of 36% [44]. This aligns with the individual domain scores, with the American Academy of Family Physicians publication scoring lowest in three domains (Supplementary Table S1). Considering guidelines published by the American Cancer Society, all guidelines published from 2015 onwards scored at least 69% (2015 publication on colorectal cancer = 73% [32]; 2016 publication on head and neck cancer = 69% [33]; 2016 ACS/ASCO publication on breast cancer = 86% [27]; 2022 publication on nutrition and physical activity = 82% [34]; 2022 publication on nutrition, physical activity, and body weight = 72% [35]). The full results of quality appraisal using the Appraisal of Guidelines for Research and Evaluation II (AGREE II) tool are available in the Supplementary Materials, Table S1 and Figure S1.

Domain 1: Scope and Purpose (covering items 1 to 3) scored well, with a mean scaled score of 79% (Supplementary Table S1). In this domain, two guidelines achieved the maximum score of 100%: the 2016 publication by the ACS and ASCO and the 2022 publication by the European Head and Neck Society [27,45]. The lowest-scoring guideline in this domain was the 2023 publication by the American Academy of Family Physicians, rated at 42% [44]. All three items in this domain scored fairly evenly: item 1 (objectives specifically described), item 2 (health questions specifically described), and item 3 (population specifically described) averaged 75%, 78%, and 84%, respectively (Supplementary Figure S1).

Domain 2: Stakeholder Engagement (covering items 4 to 6) scored moderately poorly, with a mean scaled percentage of 59% (Supplementary Table S1). The highest-scoring guideline in this domain was the 2021 publication by the European Society for Clinical Education and Metabolism (ESPEN), with a score of 81% [39]. The lowest-rated guideline in this domain was the 2023 publication by the American Academy of Family Physicians, which received a score of 25% [44]. Items 4 (guideline development includes all relevant professionals) and 6 (target users clearly defined) scored fairly well, averaging 78% and 71%, respectively (Supplementary Figure S1). However, item 5 (views and preferences of target population) scored much lower, averaging 27%.

Domain 3: Rigour of Development (covering items 7 to 14) scored moderately poorly, with a mean scaled score of 59% (Supplementary Table S1). The highest-rated score in this domain was held by the 2016 publication by ASCO and the ACS, with a rating of 91% [27]. The poorest rating in this domain was a scaled average of 18%, reported for the 2015 ASCO publication [36]. The first six items in this domain (systematic evidence search, clear selection criteria, strengths and limitations of evidence described, methods for formulating recommendations clearly described, and explicit linkage between recommendations and evidence) all scored above 58% on average (Supplementary Figure S1). However, item 13 (external guideline review before publication) averaged 44%, and item 14 (update procedure provided) averaged 22%, the lowest of any item in the AGREE II tool.

Domain 4: Clarity of Presentation (items 15–17) scored highly on the AGREE II tool, with a mean scaled score of 80% (Supplementary Table S1). In this domain, 17 of 22 articles scored 75% or above. The best-rated article for this domain was the 2016 publication by the ACS and ASCO, with a scaled score of 94% [27]. The lowest-rated guidelines in this domain were the 2023 American Academy of Family Physicians publication and the 2015 ASCO publication, both of which scored 58% [36,44]. All three items in this domain (specificity of recommendations, description of different treatment options, and identifiability of key recommendations) averaged at least 78%, scoring 85%, 78%, and 78%, respectively (Supplementary Figure S1).

Domain 5: Applicability (items 18–21) is the lowest-scoring domain in the AGREE II tool, with a scaled mean percentage of 39% (Supplementary Table S1). Fourteen of the 22 guidelines assessed scored below 50%. The best-rated article for this domain was the 2019 ASCO publication, with a scaled score of 77% [37]. The worst-scored publication was the 2003 guideline by the American Cancer Society, with a scaled percentage of 6% [29]. Every item in this domain (facilitators and barriers to application described; advice and tools for practical use provided; resource implications considered; and monitoring and auditing criteria presented) had a mean score below 50%, averaging 47%, 43%, 28%, and 39%, respectively (Supplementary Figure S1).

Domain 6: Editorial Independence (covering items 22 and 23) was the highest-scoring domain in the AGREE II instrument, with a mean scaled percentage of 81% (Supplementary Table S1). In this domain, eight publications achieved the maximum score of 100%: the 2014, 2015, and 2016 guidelines by the ACS [31,32,33]; the 2022 ACS publication titled “American Cancer Society nutrition and physical activity guideline for cancer survivors” [34]; the 2016 ACS/ASCO publication [27]; both the 2015 and 2019 guidelines published by ASCO [36,37]; and the 2017 publication by ESPEN [38]. The lowest scaled percentage, 46%, was observed for the 2018 paper by the European Society for Medical Oncology (SEOM) and the 2024 WCRF publication [43,46]. Item 22 (guideline content not influenced by funding body) ranked highly on average, scoring 92%, while item 23 (competing interests recorded and addressed) averaged 69% (Supplementary Figure S1).

#### 3.3.2. AGREE-REX Supplement

Within the AGREE-REX (Recommendation EXcellence) supplement, the scaled average percentage for all guidelines was 47% (Table 2). The item with the highest mean scaled score was Item 1: Evidence, (which considers the quality, scrutiny, and use of suitable evidence), which averaged 72% (range = 25–100%; Supplementary Table S2). The items with the lowest scaled average were items 5 and 6 (Values and Preferences of Patients and Populations; and Values and Preferences of Policy/Decision-Makers), which both averaged 25 (range = 0–75% and range = 0–83% respectively). Within the AGREE-REX framework, the highest-rated guideline was the 2016 publication created by the collaboration of ACS and ASCO (“American Cancer Society/American Society for Clinical Oncology Breast Cancer Survivorship Care Guideline”), scoring 78% [27]. The lowest rated guideline was the 2001 American Cancer Society Publication (“Nutrition during and after cancer treatment: a guide for informed choices by cancer survivors”), with a scaled score of 23% [13]. The supplementary result considering whether the reviewers would recommend a guideline for use in its appropriate context was also recorded: ten guidelines achieved a vote of “yes”; eight guidelines achieved a vote of “yes, with modifications”; and four guidelines achieved a vote of “no” (Table 2).

#### 3.3.3. Comparison of AGREE II and AGREE-REX Results

Considering the results of both tools (AGREE II and AGREE-REX), the scaled mean percentage differs, with the guidelines averaging 63% (range = 36–86%) when scored using the AGREE II tool, but just 47% (range = 25–72%) when rated using the AGREE-REX supplement (Table 2). This is perhaps an indicator of differences in exactly what the tools aim to capture, with the AGREE II tool assessing methodological quality and rigour, while the AGREE-REX supplement addresses clinical considerations such as credibility and implementability [20,21]. The 2016 publication by the ACS and ASCO ranked most highly on both tools (AGREE II = 86%; AGREE-REX = 78%) [27]. With similar convergence, four clinical practice guidelines ranked in the bottom five scores for both the AGREE II and AGREE-REX tools: the 2023 American Academy of Family Physicians publication, the 2001 and 2003 ACS publications, and the 2018 SEOM publication [13,29,44,46].

Six of the 22 clinical practice guidelines that met eligibility criteria are older guidelines that have been superseded by another guideline also within eligibility criteria: the 2001, 2003 and 2006 guidelines published by the American Cancer Society have been superseded by the 2022 ACS guidelines by Rock et al. [13,29,30,34]; the 2017 publication by ESPEN has been superseded by the 2021 ESPEN guidelines [38,39]; and the 2014 and 2022 NCCN publications have been superseded by the 2025 NCCN clinical practice guidelines [40,41,42]. The inclusion of these older superseded guidelines only had a limited impact on the mean scaled scores for both the AGREE II and the AGREE-REX tools. The average AGREE II scaled score for all guidelines was two percentage points lower than the mean for only current guidelines (mean = 65% vs. 63%), with a similar pattern in the average for the AGREE-REX tool (all guidelines mean = 47%; current guidelines mean = 50%).

Considering the magnitude of the variance between tools, two guidelines had a percentage point difference between overall scores of below 5%: the 2015 ASCO publication on obesity and cancer (AGREE II = 37%; AGREE-REX = 32%) and the 2020 WCRF/AICR publication (AGREE II = 53%; AGREE-REX = 51%) (Table 2) [28,36]. The largest percentage point difference in scoring was a 29% difference in the 2022 publication by the European Head and Neck Society (“European Head and Neck Society recommendations for head and neck cancer survivorship care” [45]), with a scaled percentage of 74% in the AGREE II, and 45% in the AGREE-REX tool. Within the AGREE-REX supplement, the European Head and Neck Society publication scored most poorly in sections that scored Values and Preferences of Guideline Developers (item 7); and Local Application and Adoption (item 9), indicating that this guideline was developed with a higher degree of methodological rigour (as scored in the AGREE II tool), but a lower emphasis on transparency of authorship and on tools for practical implementation of recommendations (Supplementary Tables S1 and S2) [45]. These findings demonstrate that using the AGREE II tool along with the AGREE-REX supplement yields unique data and insights for clinicians and researchers compared with using the AGREE II tool alone.

### 3.4. Nutrition Recommendations for People Living After Cancer

#### 3.4.1. Overall Recommendations

To assist with research and clinical decision-making comparisons between different clinical practice guidelines, the recommendations within the included publications that specifically consider nutrition and/or dietary management were extracted and analysed. Within the 22 publications, commonalities in types of recommendations provided were noted (Table 3): weight management to prevent or treat overweight and obesity; consumption of more fruits, vegetables, and wholegrains; limitation of intake of processed foods; limitation of fat and/or saturated fat consumption; limitation of intake of meat products and/or red meat products; limitation of alcohol consumption; and recommendation of strategies for symptom management. Regarding weight management, 21 publications provided recommendations to prevent or treat overweight and obesity, with only one article not providing a recommendation in this area (the 2019 ASCO publication [37]). Nineteen publications each recommended increased consumption of fruits, vegetables, and wholegrains, and limited alcohol consumption. Thirteen publications each recommended limiting fat and/or saturated fat in the diet, and limiting the consumption of meat and/or red meat. Limiting the intake of processed foods was recommended by eleven articles. Seven of the 22 articles proposed strategies for symptom management.

Considering the number of common recommendations in the articles, six clinical practice guidelines had recommendations for six of the seven categories (the 2003, 2006, 2014, and 2015 ACS publications; the 2014 publication by the NCCN; and the 2020 WCRF/AICR publication [28,29,30,31,32,40]) (Table 3). In contrast, the 2015 ASCO publication considered only weight management, providing recommendations for a single common category [36].

#### 3.4.2. Recommendations for People Living After Breast Cancer

Six clinical practice guidelines provided recommendations specifically for people living after breast cancer, the second most commonly considered cancer type, alongside head and neck cancer (Table 4). When comparing these dietary guidelines, some areas align in recommendations; however, some topics have different guidance. Many of the most commonly considered topics have consensus among different guidelines: all recommend avoiding obesity and maintaining a healthy body weight as well as emphasising the importance of vegetable and wholegrain intake, indicative of the strength of evidence and expert consensus on these topics. However, there are variations in recommendations for other topics, such as alcohol intake and consumption of soy products. The 2001 publication by the American Cancer Society and the 2024 World Cancer Research Fund publication both recommend limiting or avoiding alcohol intake due to its status as a carcinogen [13,43], whereas the 2003 publication by the American Cancer Society indicates moderate consumption may be beneficial for heart health following the results of a narrative review of observational evidence [29], and both the 2006 and 2022 publications by the American Cancer Society indicate this is a complex topic without consensus [30,34]. Similarly, the consumption of soy products is contentious due to their isoflavone content, with some guidelines theorising that excess consumption may increase oestrogen levels and increase risk of oestrogen-receptor-positive breast cancer recurrence [13,29,30,47], and others indicating that the antioxidant properties may reduce risk of cancer recurrence [34,48].

The level of evidence underpinning the recommendation for or against consumption of soy is varied: the first guideline (2001 ACS guideline) referenced 11 primary research articles considering the role of soy, soy isoflavones, or specific soy isoflavone Genistein, with most research describing in vitro outcomes of exposure on human or mouse cancer cells [13]. The 2003 ACS guideline strengthens this, considering the research through one pre/post-intervention study to determine the effect of high intake of soy protein on oestrogen in pre- and postmenopausal women, and three systematic reviews considering epidemiological and in vitro studies; however, some of this research lacks a direct link to cancer outcomes, and no systematic reviews describe any quality assessment process to underpin their results [29]. In contrast, the 2022 ACS publication titled “American Cancer Society nutrition and physical activity guideline for cancer survivors” had a stronger evidence base, considering meta-analyses of the association between soy protein or estimated soy isoflavone intake and reporting of relative risk of overall mortality [34].

Much of the data on the impact of isoflavone consumption on breast cancer prognosis and recurrence are the result of observational and epidemiological studies, making determinations of causation challenging [43,49].

#### 3.4.3. Recommendations for People Living After Colorectal Cancer

All guidelines specifically referencing people living after colorectal cancer share a generally high level of consensus; however, most guidelines do not make recommendations for all of the same key topics, despite all being published by the American Cancer Society (Table 5). Only four of the five guidelines reference the connection between red or processed meat intake and colorectal cancer risk and potential recurrence [29,30,32,34]. Supplementation is referenced in only three guidelines: the 2001 publication suggests potential benefits of selenium supplementation based upon an unexpected outcome of reduced colorectal cancer diagnosis in a trial designed to affect skin cancer incidence [13]; and the 2003 and 2006 publications suggest potential benefits of calcium supplementation based upon research into calcium carbonate supplementation as a preventative factor for the development of noncancerous colorectal adenomas, with the rationale that reducing the likelihood of colorectal adenomas may reduce the chance of developing colorectal cancer from progression of colorectal adenoma [29,30]. Similarly, only three guidelines referenced the potential benefit of limiting alcohol intake [29,30,34] and the benefit of limiting saturated fat consumption [13,29,32]. The specific guidance that healthcare providers offer depends heavily on which guidelines they follow, so important topics, such as fat intake or supplement recommendations, may be overlooked if those issues are not addressed in a particular guideline. As a result, the advice patients receive can vary significantly, potentially leaving some concerns unaddressed.

#### 3.4.4. Recommendations for People Living After Prostate Cancer

Many of the guidelines for people living after prostate cancer place strong emphasis on areas such as saturated fat intake, eating vegetables and wholegrains, and calcium and vitamin D intake (Table 6). There was a fairly high level of consensus among guidelines that consider these areas, which is perhaps unsurprising given that they were all developed by the American Cancer Society. Surprisingly, the most recent guideline (published in 2022) has fewer concrete recommendations than earlier publications, perhaps indicating the increasing complexity of providing nutritional guidelines that align with a growing evidence base [34]. Furthermore, some of the early guidelines (published in 2001, 2003, and 2006) have unclear recommendations, such as the potentially harmful impact of “food from animal sources” [13,29,30]; whether this is considering only meat products or all animal products is unclear, and the nutrition implications of severely limiting or removing these food groups from the diet are serious when no alternative options are provided [50].

#### 3.4.5. Recommendations for People Living After Head and Neck Cancer

Six clinical practice guidelines provide specific recommendations for people living after head and neck cancer, equal to or more than any other cancer site, alongside breast cancer (Table 7). Many guidelines provided recommendations for symptom management, with five publications (the 2001, 2003, 2006, and 2016 ACS guidelines, and the 2022 European Head and Neck Society publication) providing guidance on topics like limiting irritation of the oropharynx and oesophagus during eating and drinking, reducing the likelihood of reflux or regurgitation, and using texture-modified foods as required [13,29,30,33,45]. All six articles provide recommendations aimed at improving survival and quality of life and reducing the risk of cancer recurrence, with the most common recommendations including maintaining a healthy weight, increasing intake of energy- and protein-rich foods, and reducing alcohol consumption.

#### 3.4.6. Recommendations for People Living After Other Cancer Types

Five of the 22 clinical practice guidelines provided specific reference to nutritional recommendations for people living after other cancer types [13,26,29,30,34]. The most commonly referenced other cancer type was lung cancer, which was considered in four guidelines, with differing recommendations: three publications (the 2001, 2003, and 2006 ACS publications) recommended increased fruit and vegetable intake to reduce the risk of cancer recurrence; however, the 2022 ACS publication indicated that there was insufficient evidence to support this recommendation specifically for people living after lung cancer [13,29,30,34]. In this category, the 2001 ACS publication also recommended limiting nutrient intake through dietary or supplementary forms to below the upper limit for each nutrient, as there was some evidence to suggest that extremely high beta carotene intake may be associated with lung cancer recurrence [13]. Upper gastrointestinal and upper aerodigestive cancers were considered cumulatively three times, with guidance on each occasion recommending alignment with the head and neck cancer recommendations provided by each of these articles (described above in Table 7) [29,30,34]. Digestive and/or gastric cancers were referenced by three articles, all with differing recommendations: the 2001 ACS publication recommended strategies like eating slowly and limiting high-sugar foods to reduce reflux and rapid transit; the 2022 ACS publication recommended that people living after digestive cancers follow general guidelines (described above in Table 3); and the 2021 French intergroup guidelines specifically stated they had “no recommendations” for this group [13,26,34]. Two guidelines provided recommendations for people living after haematologic cancers, with the 2006 ACS publication recommending weight management to prevent overweight and obesity and potentially reduce cancer recurrence risk, and the 2022 ACS publication recommending limiting alcohol consumption with the same rationale [30,34]. Finally, the 2022 ACS publication also provided brief recommendations for adults living after childhood cancers, recommending following a “healthy diet” for general wellbeing [34].

## 4. Discussion

This review assessed the methodological quality and clinical applicability of clinical practice guidelines for the nutritional management of people living after cancer using the AGREE II and AGREE-REX tools [20,21]. The included guidelines had a higher mean overall score in the AGREE II instrument than the AGREE-REX supplement (Table 2). Ten guidelines were recommended for use in the appropriate context, with the remaining twelve either being recommended if modified or not recommended at all.

The average highest scored domain using the AGREE II tools was Editorial Independence closely followed by Clarity of Presentation and Scope and Purpose. These domains are important as they address areas that are essential for the trust in and use of the guideline, including the overall objectives, health questions covered, population to whom the guideline is applicable, specificity of recommendations, and transparency around influence of funding and competing interests on the guideline development [20]. In contrast, the domain related to Applicability (Domain 5 = 39%, range = 6–77%) was less clearly considered. In particular, potential resource implications of applying the recommendations were rarely reported (Supplementary Figure S1). Resource considerations for nutritional recommendations should take into account the limitations and challenges that the target population may face in attempting to implement recommendations: “healthy” foods (as recommended by 21 of 22 resources) [13,26,27,28,29,30,31,32,33,34,35,36,38,39,40,41,42,43,44,46] are often less available and affordable [51]; “time-related barriers” to healthy eating impact time-poor groups [52]; and dietary supplements (recommended for consideration by five articles) can prove expensive, especially in lower-income populations [13,29,30,45,53]. Criteria for monitoring or auditing the application of recommendations were also often overlooked: the inclusion of these criteria for determining the application of guideline recommendations can further help clinicians determine the effectiveness of and methods for implementation of clinical practice guidelines [20]. Consideration of policies for updating the guidelines also received a lower average score, potentially leaving researchers and clinicians working with older materials that do not reflect the most current evidence [54]. For current and future clinical practice guideline developers and organisations, applicability should be considered a primary requirement for the development of clinical practice guidelines in this area: tools and resources for patients and clinicians (e.g., online/interactive tools or frameworks for implementation and monitoring; shortened messaging for quick daily reference; and patient resources, handouts, or practical examples) can assist with the practicality and time-effectiveness of clinical practice guideline implementation. Specific description of differing priorities for clients and clinicians, as well as consideration of facilitators and barriers was rare in the included guidelines; consideration of these factors as central requirements in future clinical practice guidelines would increase the implementation of the recommendations, as many nonadherent clinicians report inflexibility and impracticality to be the rationale behind their reluctance to use existing guidelines [55]. There is also some evidence to suggest many clinical practice guidelines are not pilot-tested prior to full dissemination: pilot-testing in the care delivery settings for which the resource is designed can identify limitations and oversights in the practical applicability to resolve prior to release, and should be considered in future publications [1].

Inclusion of Views and Preferences of the Target Population (i.e., people living after cancer) also scored poorly on average (item 5 = 27%), with many guidelines failing to have any patient representatives (Supplementary Table S3). While collaboration with the target population to develop clinical practice guidelines can present challenges (including recruitment, lack of representation, lack of familiarity of medical and scientific terms, and concerns of bias) [56], the inclusion of the target population directly in guideline development has been established as an “integral” step to ensure the guidelines are person-centred, align with stakeholder priorities, and provide trustworthy guidance [57,58]. Guideline development that does not include the target population as essential stakeholders risks producing guidelines that are impractical, misaligned with the reality of the patient experience, and poorly implemented; this negatively impacts clinicians and healthcare systems that rely on the guidance from these publications, as the care and counselling they provide could become unachievable, untrustworthy, and oblivious to the lived experience of their clients. Patient involvement in guideline development has become increasingly understood as crucial as research continues to evolve, and indeed, many guideline development manuals consider patient involvement as an essential tool for resource creation [57]. This trend is apparent in the included guidelines to an extent, with guidelines published in or prior to 2015 averaging 15% in the Target Population Views And Preferences item, while guidelines published after 2015 averaged 32%; however, the low means for both groups indicate an ongoing requirement for this to be considered and prioritised in the update of existing guidelines and the development of new clinical practice guidelines (Supplementary Table S1). There is also evidence that engagement with patients in the development of guidelines is practical and possible, with a 2022 scoping review identifying 47 guidelines that prioritised patient involvement, including 5 guidelines relating to cancer, published by organisations in countries including Canada, the Netherlands, Australia, the United States, Germany, Italy and Spain [59]. The most common roles for target population involvement in these were in identifying relevant questions, reviewing drafts of the clinical practice guidelines, and assisting with the development of a matched patient resource.

The findings of this review are consistent with similar systematic reviews of clinical practice guidelines by Zhou et al. in nutritional risk screening for cancer patients [60] and by Noyahr et al. and Padilla et al., which both consider nutrition in critically ill adults [22,61]. These papers all reported the highest scores in the domains of Scope and Purpose and Clarity of Presentation and lowest scores in Applicability, as were determined in this review (Supplementary Table S1). Low-rated items among different reviews were also similar to the findings of this review: Noyahr et al. and Padilla et al. both reporting infrequent patient involvement in the guidelines evaluated and thus low consideration of target population values and preferences [22,61], while Zhou et al. reported that no guidelines assessed had any involvement from the target population (Supplementary Figure S1) [60]. In line with the results of this study, Zhou et al. also found resource implications to be the lowest scored of any individual item, with many clinical practice guidelines not addressing this topic at all.

The AGREE-REX supplement scored an average of 47% (range = 23–78%), which was lower than the AGREE II tool, indicating that guidelines were more likely to prioritise methodology than clinical applicability (Table 2). The highest ranked item was Evidence (item 1 mean = 72%, range = 25–100%), which was often high given that most clinical practice guidelines were based on a literature review and/or expert consensus, and that many clinical practice guidelines included evidence grading in the decision-making process. (Supplementary Table S2). Values and Preferences of Policy/Decision-Makers (item 6 mean = 25%, range = 0–83%) scored poorly on average, indicating that many guidelines did not consider what would be important to this group. Given that many guidelines have poor adoption by clinicians, with some reports estimating that clinicians adhere to relevant guidelines as little as 27% of the time [62], and that even practitioners working in cancer care adhere to clinical practice guidelines as little as 29% of the time [63], guidelines should be aiming to improve the ease of their own implementation. With 19 of 22 guidelines scoring below 50% in this item [13,26,27,29,30,31,32,33,34,35,36,39,40,41,42,43,44,45,46], most guidelines have not prioritised consideration of the needs and preferences of policy- and decision-makers, despite reports that state that “environmental” factors controlled by these groups like employer support, policy development, and process alignment can facilitate greater adherence [64,65,66].

When considering the common recommendations provided by the clinical practice guidelines, no single guideline provided specific guidance on all common areas of importance for general cancer recommendations or for any of the major cancer types, with many guidelines providing nonspecific advice like “consider nutrition support”, and “reduce food from animal sources” (Table 4, Table 5, Table 6 and Table 7). While a degree of uncertainty is common in research [67], ambiguity and complexity are associated with reduced guideline adherence among clinicians, thereby diminishing the publication’s effectiveness [65].

Decision-making by clinicians and researchers on the choice and suitability of nutrition clinical practice guidelines for people living after cancer is inherently complex, and this review is designed to provide a framework for making appropriate selections rather than definitive recommendations on which guideline to adopt. The most highly rated guideline according to the AGREE II and AGREE-REX instruments is the ACS/ASCO publication on breast cancer survivorship care (Table 2); however, this publication is only applicable to people living after breast cancer and does not provide recommendations for other cancer types [27]. This guideline is also ten years old at the time of this review’s publication, which may be considered out of date by clinicians and organisations [54]. Other guidelines like the 2025 NCCN publication and the 2023 publication by the American Academy of Family Physicians were published more recently [44]; however, these guidelines score more poorly in the quality assessment tools than some of the other guidelines published 5–10 years ago. The 2022 American Cancer Society publication on nutrition and physical activity guidelines is perhaps the most commonly considered guideline [34], with research measuring compliance and demonstrating an association between improved adherence and improved symptoms, inflammatory markers, and survival rates [68,69,70,71,72]. Given the evidence to support the effectiveness of this guideline, some clinicians may feel more comfortable using this publication.

In assessment of full-text eligibility, 16 guidelines that otherwise met inclusion criteria were eliminated due to meeting the exclusion criteria for not providing specificity on nutrition recommendations. These guidelines provided nonspecific advice such as eating a healthy diet or recommending that dietitians provide clinical guidance, which is largely insufficient given that these clinical practice guidelines are designed to guide the practitioner. This is perhaps an indicator of an undervaluation of the role of nutrition and dietetics in the care of people living after cancer in the wider field of comprehensive cancer care, with an assumption that this area requires less specificity or that dietetics practice in this area is safe and effective, relying on the clinical judgement of the nutrition professional without requiring guidance. Given that some groups like people living after head and neck cancer are prone to severe nutrition complications for months and even years after treatment, this perspective of the limited efficacy or inherent safety of nutrition therapy could be very damaging [73]. There is also evidence to suggest that people living after head and neck cancer benefit strongly from specific continued treatment beyond the bounds of the “active” phase of disease, with a large retrospective cohort study of over 38,000 people living with and after head and neck cancer determining that prolonged persistence of medical nutrition therapy (≥7-month duration vs. 1–3- and 4–6-month duration) was associated with significantly longer overall survival [74].

The strengths of this review include the use of valid and reliable tools, specifically designed to assess the quality of clinical practice guidelines. All authors involved in the quality appraisal and review of included clinical practice guidelines have sufficient proficiency to critically assess nutrition guidelines, and all authors hold postgraduate degrees in nutrition and/or dietetics. This publication holds a practical focus for clinicians and researchers: older guidelines that have been superseded by newer releases were included alongside the newer release to assist with clinical and research considerations for the reader, as these older guidelines have potentially been integrated into decision-making standards for healthcare organisations and providers, as well as forming the foundation of research into effectiveness of guideline adherence on survival [69,75].

Limitations of this review include the methodology used to ensure consistency of screening and quality analysis between reviewers: while rater calibration can improve inter-rater reliability, using multiple reviewers for the same role in screening and quality analysis allows for persistent uncertainty [76]. Considering the practical use of this review, the AGREE II and AGREE-REX instruments do not provide cutoff points to define groupings such as high-, moderate-, and low-quality guidelines, meaning that the quality appraisal results can be used for comparison but not for definitive decision-making without more holistic consideration of the guideline itself. Some organisations had older guidelines that have been superseded by newer release included in the dataset; this resulted in some organisations (ACS, ESPEN, and NCCN) being overrepresented in the dataset and a minor sway in the results reported, with the inclusion of superseded guidelines resulting in the AGREE II scaled score mean being two percentage points lower and the AGREE-REX scaled score mean being three percentage points lower than if these guidelines had been excluded. Several articles were also deleted by the guideline developer during the period between title and abstract screening and full-text screening (as shown in Figure 1), potentially resulting in the omission of relevant resources. This review also considered only guidelines available both online and in English, limiting its scope and potentially introducing English-language bias. The included articles were also geographically skewed, with 16 of the 22 guidelines written by organisations in the United States. While this is reflective of the literature in this area, it could introduce a Western-centric tendency into the results.

## 5. Conclusions

This review aimed to assess the quality of clinical practice guidelines providing guidance on nutrition for people living after cancer and to identify and describe themes in their recommendations. Ten of the 22 clinical practice guidelines were of sufficient quality that this review would recommend them for use in their appropriate contexts. While included clinical practice guidelines for nutritional management after cancer demonstrate areas of strong methodological quality, particularly in Clarity, Editorial Independence, and Scope, they frequently lack detailed consideration of Applicability, resource implications, and Stakeholder Involvement. The differences in recommendations among guidelines for the management of the same cancer types highlighted the evolving nature of research and the challenges clinicians face in selecting evidence to guide practice. Continued efforts to systematically involve stakeholders and to provide clearer, more specific guidance will be crucial to enhancing the relevance, adoption, and impact of nutritional recommendations for clinicians working with people living after cancer.

## Figures and Tables

**Table 1:** Characteristics of clinical practice guidelines.

Organisation	Year Published	Guideline Title	Country	Authors	Source of Evidence	Populations Considered	Types of Cancer Referenced
American Academy of Family Physicians	2023	Care of Cancer Survivors: Nutrition and Physical Activity [44]	United States	Poling	Unspecified literature review	People with cancer, people living after cancer	Not specified
American Cancer Society (ACS)	2001	Nutrition during and after cancer treatment: a guide for informed choices by cancer survivors [13]	United States	Brown, Byers, Thompson et al.	Literature “compilation and review”	People undergoing cancer treatment, people living after cancer, people with advanced cancer	Breast cancer, prostate cancer, lung cancer, head and neck cancers, gastric cancer, colorectal cancer
	2003	Nutrition and physical activity during and after cancer treatment: an American Cancer Society guide for informed choices [29]	United States	Brown, Byers, Doyle et al.	Expert consensus with some evidence grading	People undergoing cancer treatment, people living after cancer	Breast cancer, colorectal cancer, lung cancer, prostate cancer, upper gastrointestinal, head and neck cancers
	2006	Nutrition and physical activity during and after cancer treatment: an American Cancer Society guide for informed choices [30]	United States	Doyle, Kushi, Byers et al.	Expert consensus	People undergoing cancer treatment, people in recovery from cancer treatment, people living after cancer, people with advanced cancer	Breast cancer, colorectal cancer, hematologic cancer, lung cancer, prostate cancer, upper gastrointestinal, head and neck cancers
	2014	American Cancer Society Prostate Cancer Survivorship Care Guidelines [31]	United States	Skolarus, Wolf, Erb et al.	Expert consensus	People living after prostate cancer	Prostate cancer
	2015	American Cancer Society Colorectal Cancer Survivorship Care Guidelines [32]	United States	El-Shami, Oeffinger, Erb et al.	Expert consensus of literature (not otherwise specified)	People living after colorectal cancer	Colorectal cancer
	2016	American Cancer Society Head and Neck Cancer Survivorship Care Guideline [33]	United States	Cohen, LaMonte, Erb et al.	Systematic review and expert consensus	People living after head and neck cancer	Head and neck cancer
	2022	American Cancer Society nutrition and physical activity guidelines for cancer survivors [34]	United States	Rock, Thomson, Sullivan et al.	Large-scale systematic review	People living after cancer, people with “stable disease”	Breast cancer, cancers of the upper aerodigestive and digestive system (oesophageal, colorectal, gastric, oropharyngeal, and pancreatic cancers), genitourinary cancers (prostate cancer, kidney cancer, bladder cancer), gynaecologic cancers, lung cancer, haematological cancers, childhood cancers
	2022	Nutrition, Physical Activity, Body Weight and Cancer Survivorship [35]	United States	Not listed	Literature review (not otherwise specified)	People with cancer, people living after cancer	Not specified
American Cancer Society/American Society of Clinical Oncology (ACS/ASCO)	2016	American Cancer Society/American Society of Clinical Oncology Breast Cancer Survivorship Care Guideline [27]	United States	Runowicz, Leach, Henry et al.	Systematic review and expert consensus	People living after breast cancer	Breast cancer
American Society of Clinical Oncology (ASCO)	2015	American Society of Clinical Oncology Position Statement on Obesity and Cancer [36]	United States	Ligibel, Alfano, Courneya et al.	Summary of other publications by ASCO	People at risk of cancer, people with cancer, people living after cancer	Not specified
	2019	Management of Osteoporosis in Survivors of Adult Cancers With Nonmetastatic Disease: ASCO Clinical Practice Guideline [37]	United States	Shapiro, Van Poznak, Lacchetti et al.	Expert consensus	People with nonmetastatic cancer, people living after cancer	Not specified
European Head and Neck Society	2022	European Head and Neck Society recommendations for head and neck cancer survivorship care [45]	Europe (various)	Verdonck-de Leeuw, Dawson, Licitra et al.	Adolopment of American Cancer Society Head and Neck Guidelines	People living after head and neck cancer	Head and neck cancer
European Society for Clinical Nutrition and Metabolism (ESPEN)	2017	ESPEN guidelines on nutrition in cancer patients [38]	Europe (various)	Arends, Bachmann and Baracos et al.	Review of evidence using GRADE method	People with cancer, people living after cancer	Not specified
	2021	ESPEN practical guideline: Clinical Nutrition in cancer [39]	Europe (various); Australia and Canada	Muscaritoli, Arends, Bachmann et al.	Expert consensus (incl patient representatives)	Cancer patients, people living after cancer, people with advanced cancer	Not specified
European Society for Medical Oncology (SEOM)	2018	SEOM clinical guidelines on nutrition in cancer patients (2018) [46]	Spain	de las Peñas, Majem, Pérez-Altozano et al.	Expert consensus and evidence grading using USPSTF method	People with cancer, people living after cancer, people with advanced cancer	Not specified
National Comprehensive Cancer Network (NCCN)	2014	Survivorship: nutrition and weight management, Version 2.2014 Clinical practice guidelines in oncology [40]	United States	Denlinger, Ligibel, Are et al.	Expert consensus, literature review with evidence grading	People living after cancer	Not specified
	2022	NCCN Guidelines Insights: Survivorship, Version 1.2022 [41]	United States	Sanft, Day, Peterson et al.	Literature review with evidence grading	People with cancer, people living after cancer	Not specified
	2025	NCCN Guidelines Insights: Survivorship, Version 2.2025 [42]	United States	Sanft, Day, Ansbaugh et al.	Expert consensus (unspecified)	People with cancer, people living after cancer	Not specified
SNFGE ^a^, FFCD ^b^, GERCOR ^c^, UNICANCER ^d^, SFCD ^e^, SFED ^f^, SFRO ^g^, ACHBT ^h^, AFC ^i^, SFP-APA ^j^, SFNCM ^k^, AFSOS ^l^	2021	Nutrition and physical activity: French intergroup clinical practice guidelines for diagnosis, treatment and follow-up (SNFGE, FFCD, GERCOR, UNICANCER, SFCD, SFED, SFRO, ACHBT, AFC, SFP-APA, SFNCM, AFSOS) [26]	France	Neuzillet, Anota, Foucat et al.	Expert consensus (unspecified)	People with cancer, people living after cancer	Digestive cancers
World Cancer Research Fund (WCRF)	2024	Diet, nutrition, physical activity and body weight for people living with and beyond breast cancer [43]	International (unspecified)	Krebs, Weijenberg, Baskin et al.	Expert consensus and evidence grading	People with breast cancer, people living after breast cancer	Breast cancer
World Cancer Research Fund/American Institute for Cancer Research (WCRF/AICR)	2020	The World Cancer Research Fund/American Institute for Cancer Research Third Expert Report on Diet, Nutrition, Physical Activity, and Cancer: Impact and Future Directions [28]	United States	Clinton, Giovannucci, Hursting et al.	Expert consensus and evidence grading as part of CUP project	People at risk of cancer, people with cancer, people living after cancer	Not specified

^a^ Société Nationale Française de Gastroentérologie; ^b^ Fédération Francophone de Cancérologie Digestive; ^c^ Groupe Coopérateur multidisciplinaire en Oncologie; ^d^ Fédération Nationale des Centres de Lutte Contre le Cancer; ^e^ Société Française de Chirurgie Digestive; ^f^ Société Française d’Endoscopie Digestive; ^g^ Société Française de Radiothérapie Oncologique; ^h^ Association de Chirurgie Hépato-Bilio-Pancréatique et Transplantation; ^i^ Association Française de Chirurgie; ^j^ Société Française des Professionnels en Activité Physique Adaptée; ^k^ Société Francophone de Nutrition Clinique et Métabolisme; ^l^ Association Francophone pour Soins Oncologiques de Support.

**Table 2:** Overall AGREE II and AGREE-REX scores.

Organisation	Year	Title	AGREE II Score (Range) *	AGREE-REX Score (Range) *	Recommendation for Use in Suitable Context?
American Academy of Family Physicians	2023	Care of Cancer Survivors: Nutrition and Physical Activity [44]	36% (0–100%)	24% (8–50%)	No
American Cancer Society (ACS)	2001	Nutrition during and after cancer treatment: a guide for informed choices by cancer survivors [13]	42% (0–100%)	23% (8–42%)	No
	2003	Nutrition and physical activity during and after cancer treatment: an American Cancer Society guide for informed choices [29]	47% (0–100%)	30% (8–58%)	Yes, with modifications
	2006	Nutrition and physical activity during and after cancer treatment: an American Cancer Society guide for informed choices [30]	52% (0–100%)	27% (8–58%)	No
	2014	American Cancer Society Prostate Cancer Survivorship Care Guidelines [31]	59% (8–100%)	41% (17–83%)	Yes, with modifications
	2015	American Cancer Society Colorectal Cancer Survivorship Care Guidelines [32]	73% (8–100%)	49% (8–83%)	Yes
	2016	American Cancer Society Head and Neck Cancer Survivorship Care Guideline [33]	69% (8–100%)	50% (8–83%)	Yes
American Cancer Society (ACS)	2022	American Cancer Society nutrition and physical activity guidelines for cancer survivors [34]	82% (25–100%)	55% (25–92%)	Yes
	2022	Nutrition, Physical Activity, Body Weight and Cancer Survivorship [35]	72% (8–100%)	53% (17–83%)	Yes
American Cancer Society/American Society for Clinical Oncology	2016	American Cancer Society/American Society of Clinical Oncology Breast Cancer Survivorship Care Guideline [27]	86% (50–100%)	78% (42–100%)	Yes
American Society for Clinical Oncology (ASCO)	2015	American Society of Clinical Oncology Position Statement on Obesity and Cancer [36]	37% (0–100%)	32% (0–50%)	No
	2019	Management of Osteoporosis in Survivors of Adult Cancers With Nonmetastatic Disease: ASCO Clinical Practice Guideline [37]	82% (17–100%)	62% (25–92%)	Yes, with modifications
European Head and Neck Society	2022	European Head and Neck Society recommendations for head and neck cancer survivorship care [45]	74% (0–100%)	45% (8–92%)	Yes, with modifications
European Society for Clinical Nutrition and Metabolism (ESPEN)	2017	ESPEN guidelines on nutrition in cancer patients [38]	85% (17–100%)	68% (8–100%)	Yes
	2021	ESPEN practical guideline: Clinical Nutrition in cancer [39]	67% (0–92%)	54% (33–75%)	Yes, with modifications
European Society for Medical Oncology (SEOM)	2018	SEOM clinical guidelines on nutrition in cancer patients (2018) [46]	47% (0–83%)	31% (0–67%)	Yes, with modifications
National Comprehensive Cancer Network (NCCN)	2014	Survivorship: nutrition and weight management, Version 2.2014. Clinical practice guidelines in oncology [40]	60% (17–92%)	42% (8–67%)	Yes
	2022	NCCN Guidelines Insights: Survivorship, Version 1.2022 [41]	58% (8–100%)	52% (8–83%)	Yes
	2025	NCCN Guidelines Insights: Survivorship, Version 2.2025 [42]	62% (17–92%)	56% (8–100%)	Yes
SNFGE ^a^, FFCD ^b^, GERCOR ^c^, UNICANCER ^d^, SFCD ^e^, SFED ^f^, SFRO ^g^, ACHBT ^h^, AFC ^i^, SFP-APA ^j^, SFNCM ^k^, AFSOS ^l^	2021	Nutrition and physical activity: French intergroup clinical practice guidelines for diagnosis, treatment and follow-up (SNFGE, FFCD, GERCOR, UNICANCER, SFCD, SFED, SFRO, ACHBT, AFC, SFP-APA, SFNCM, AFSOS) [26]	64% (17–92%)	48% (0–75%)	Yes, with modifications
World Cancer Research Fund (WCRF)	2024	Diet, nutrition, physical activity and body weight for people living with and beyond breast cancer [43]	71% (17–100%)	65% (33–92%)	Yes
World Cancer Research Fund, American Institute for Cancer Research	2020	The World Cancer Research Fund/ American Institute for Cancer Research Third Expert Report on Diet, Nutrition, Physical Activity, and Cancer: Impact and Future Directions [28]	53% (17–92%)	51% (0–83%)	Yes, with modifications
Mean			63% (36–86%)	47% (25–72%)	

* Calculated according to the method outlined in the AGREE II manual; ^a^ Société Nationale Française de Gastroentérologie; ^b^ Fédération Francophone de Cancérologie Digestive; ^c^ Groupe Coopérateur multidisciplinaire en Oncologie; ^d^ Fédération Nationale des Centres de Lutte Contre le Cancer; ^e^ Société Française de Chirurgie Digestive; ^f^ Société Française d’Endoscopie Digestive; ^g^ Société Française de Radiothérapie Oncologique; ^h^ Association de Chirurgie Hépato-Bilio-Pancréatique et Transplantation; ^i^ Association Française de Chirurgie; ^j^ Société Française des Professionnels en Activité Physique Adaptée; ^k^ Société Francophone de Nutrition Clinique et Métabolisme; ^l^ Association Francophone pour Soins Oncologiques de Support.

**Table 3:** Overall nutrition recommendations for people living after cancer.

Organisation	Year	Title	Weight Management or Reduction of Overweight and Obesity	Consume More Fruit, Vegetables, and Wholegrains	Limit Processed Food	Limit Fat or Saturated Fat	Limit Meat or Red Meat Products	Limit Alcohol	Strategies for Symptom Management
American Academy of Family Physicians	2023	Care of Cancer Survivors: Nutrition and Physical Activity [44]	✔	✔	✔		✔	✔	
American Cancer Society (ACS)	2001	Nutrition during and after cancer treatment: a guide for informed choices by cancer survivors [13]	✔	✔		✔	✔	✔	
	2003	Nutrition and physical activity during and after cancer treatment: an American Cancer Society guide for informed choices [29]	✔	✔	✔	✔	✔	✔	
	2006	Nutrition and physical activity during and after cancer treatment: an American Cancer Society guide for informed choices [30]	✔	✔	✔	✔	✔	✔	
	2014	American Cancer Society Prostate Cancer Survivorship Care Guidelines [31]	✔	✔	✔	✔		✔	✔
	2015	American Cancer Society Colorectal Cancer Survivorship Care Guidelines [32]	✔	✔	✔	✔		✔	✔
	2016	American Cancer Society Head and Neck Cancer Survivorship Care Guideline [33]	✔	✔		✔		✔	✔
	2022	American Cancer Society nutrition and physical activity guidelines for cancer survivors [34]	✔	✔	✔			✔	
	2022	Nutrition, Physical Activity, Body Weight and Cancer Survivorship [35]	✔	✔	✔		✔	✔	
American Cancer Society/American Society of Clinical Oncology (ACS/ASCO)	2016	American Cancer Society/American Society of Clinical Oncology Breast Cancer Survivorship Care Guideline [27]	✔	✔		✔		✔	✔
American Society for Clinical Oncology (ASCO)	2015	American Society of Clinical Oncology Position Statement on Obesity and Cancer [36]	✔						
	2019	Management of Osteoporosis in Survivors of Adult Cancers With Nonmetastatic Disease: ASCO Clinical Practice Guideline [37]						✔	✔
European Head and Neck Society	2022	European Head and Neck Society recommendations for head and neck cancer survivorship care [45]	✔	✔		✔		✔	✔
European Society for Clinical Nutrition and Metabolism (ESPEN)	2017	ESPEN guidelines on nutrition in cancer patients [38]	✔	✔		✔	✔	✔	
	2021	ESPEN practical guideline: Clinical Nutrition in cancer [39]	✔	✔		✔	✔	✔	
European Society for Medical Oncology (SEOM)	2018	SEOM clinical guidelines on nutrition in cancer patients (2018) [46]	✔	✔		✔	✔	✔	
National Comprehensive Cancer Network (NCCN)	2014	Survivorship: nutrition and weight management, Version 2.2014 Clinical practice guidelines in oncology [40]	✔	✔	✔	✔	✔	✔	
	2022	NCCN Guidelines Insights: Survivorship, Version 1.2022 [41]	✔	✔	✔		✔	✔	
	2025	NCCN Guidelines Insights: Survivorship, Version 2.2025 [42]	✔	✔	✔		✔	✔	
SNFGE ^a^, FFCD ^b^, GERCOR ^c^, UNICANCER ^d^, SFCD ^e^, SFED ^f^, SFRO ^g^, ACHBT ^h^, AFC ^i^, SFP-APA ^j^, SFNCM ^k^, AFSOS ^l^	2021	Nutrition and physical activity: French intergroup clinical practice guidelines for diagnosis, treatment and follow-up (SNFGE, FFCD, GERCOR, UNICANCER, SFCD, SFED, SFRO, ACHBT, AFC, SFP-APA, SFNCM, AFSOS) [26]	✔						✔
World Cancer Research Fund (WCRF)	2024	Diet, nutrition, physical activity and body weight for people living with and beyond breast cancer [43]	✔	✔			✔		
World Cancer Research Fund, American Institute for Cancer Research	2020	The World Cancer Research Fund/ American Institute for Cancer Research Third Expert Report on Diet, Nutrition, Physical Activity, and Cancer: Impact and Future Directions [28]	✔	✔	✔	✔	✔	✔	

✔ This clinical practice guideline includes the listed recommendation; ^a^ Société Nationale Française de Gastroentérologie; ^b^ Fédération Francophone de Cancérologie Digestive; ^c^ Groupe Coopérateur multidisciplinaire en Oncologie; ^d^ Fédération Nationale des Centres de Lutte Contre le Cancer; ^e^ Société Française de Chirurgie Digestive; ^f^ Société Française d’Endoscopie Digestive; ^g^ Société Française de Radiothérapie Oncologique; ^h^ Association de Chirurgie Hépato-Bilio-Pancréatique et Transplantation; ^i^ Association Française de Chirurgie; ^j^ Société Française des Professionnels en Activité Physique Adaptée; ^k^ Société Francophone de Nutrition Clinique et Métabolisme; ^l^ Association Francophone pour Soins Oncologiques de Support.

**Table 4:** Recommendations for people living after breast cancer.

Organisation	Year	Title	Weight Management	Fat Consumption	Vegetable and Wholegrain Consumption	Elements to Limit in the Diet	Alcohol Consumption	Soy Consumption
American Cancer Society (ACS)	2001	Nutrition during and after cancer treatment: a guide for informed choices by cancer survivors [13]	Achieve and maintain healthy weight	Follow lower-fat diet	High intake	Not specified	Limit/avoid	Limit to “moderate”
	2003	Nutrition and physical activity during and after cancer treatment: an American Cancer Society guide for informed choices [29]	Achieve and maintain healthy weight	Not specified	High intake	Not specified	Moderate consumption may be beneficial	Limit to “moderate”
	2006	Nutrition and physical activity during and after cancer treatment: an American Cancer Society guide for informed choices [30]	Achieve and maintain a healthy weight	Follow lower-fat diet, limit saturated fats	High intake	Sugar, refined grains, animal products	Complex—discuss with healthcare provider	Limit to “moderate”
	2022	American Cancer Society nutrition and physical activity guideline for cancer survivors [34]	Avoid obesity	Evidence unclear	High intake	Red and processed meat, refined grains, added sugars	Evidence unclear	Consumption likely beneficial
American Cancer Society/American Society of Clinical Oncology (ACS/ASCO)	2016	American Cancer Society/American Society for Clinical Oncology Breast Cancer Survivorship Care Guideline [27]	Achieve and maintain healthy weight	Limit saturated fats	High intake	Not specified	Limit/avoid	Not specified
World Cancer Research Fund (WCRF)	2024	Diet, nutrition, physical activity and body weight for people living with and beyond breast cancer [43]	Achieve and maintain healthy weight	Not specified	High intake	Sugar-sweetened drinks, dietary supplements	Limit/avoid	Evidence unclear—dependent on personal preferences

**Table 5:** Recommendations for people living after colorectal cancer.

Organisation	Year	Title	Weight Management	Fat Consumption	Vegetable and Wholegrain Consumption	Elements to Limit in the Diet	Supplement Use	Meat Consumption
American Cancer Society (ACS)	2001	Nutrition during and after cancer treatment: a guide for informed choices by cancer survivors [13]	Avoid obesity	Limit saturated fat	High intake	Not specified	Selenium supplementation potentially beneficial	Not specified
	2003	Nutrition and physical activity during and after cancer treatment: an American Cancer Society guide for informed choices [29]	Achieve and maintain a healthy body weight	Limit saturated fat	High intake	Alcohol	Calcium supplementation potentially beneficial	Limit red and processed meats
	2006	Nutrition and physical activity during and after cancer treatment: an American Cancer Society guide for informed choices [30]	Achieve and maintain a healthy body weight	Not specified	High intake	Alcohol	Calcium supplementation potential beneficial	Limit red and processed meats
	2015	American Cancer Society Colorectal Cancer Survivorship Care Guidelines [32]	Achieve and maintain a healthy body weight	Limit saturated fat	High intake	Refined grains, “sugary desserts”	Not specified	Limit red and processed meats
	2022	American Cancer Society nutrition and physical activity guideline for cancer survivors [34]	Evidence unclear	Not specified	High intake	Alcohol, “sugary drinks”, energy-dense foods	Not specified	Limit red or processed meat

**Table 6:** Recommendations for people living after prostate cancer.

Organisation	Year	Title	Weight Management	Fat Consumption	Vegetable and Wholegrain Consumption	Elements to Limit in the Diet	Calcium and Vitamin D	Antioxidants
American Cancer Society (ACS)	2001	Nutrition during and after cancer treatment: a guide for informed choices by cancer survivors [13]	Not specified	Limit saturated fat	Not specified	“Food from animal sources”	Avoid calcium supplementation	Potential benefits from dietary lycopene and beta carotene
	2003	Nutrition and physical activity during and after cancer treatment: an American Cancer Society guide for informed choices [29]	Achieve and maintain a healthy body weight	Limit saturated fat, increase monounsaturated fats	High intake	“Food from animal sources”	Avoid high levels of calcium intake (dietary and supplements)	Potential benefit from dietary lycopene
	2006	Nutrition and physical activity during and after cancer treatment: an American Cancer Society guide for informed choices [30]	Not specified	Limit saturated fat, increase monounsaturated fats	High intake	“Food from animal sources”	At least 600 IU vitamin D, adequate/not excessive calcium (not more than 1200 mg/d)	Potential benefit from dietary lycopene
	2014	American Cancer Society Prostate Cancer Survivorship Care Guidelines [31]	Achieve and maintain a healthy body weight	Limit saturated fat	High intake	Alcohol (no more than 2 standard drinks)	At least 600 IU vitamin D, adequate/not excessive calcium (not more than 1200 mg/d)	Not specified
	2022	American Cancer Society nutrition and physical activity guideline for cancer survivors [34]	Evidence unclear	Not specified	High intake	Not specified	Not specified	Not specified

**Table 7:** Recommendations for people living after head and neck cancer.

Organisation	Year	Title	Weight Management	Energy and Protein Intake	Fat Intake	Vegetable and Wholegrain Consumption	Elements to Limit in the Diet	Texture Modifications
American Cancer Society (ACS)	2001	Nutrition during and after cancer treatment: a guide for informed choices by cancer survivors [13]	Maintain adequate weight	Maximise intake	Limit fat intake in cases of regurgitation	Continue intake	Strong flavours and acidic foods (if poorly tolerated)	As required
	2003	Nutrition and physical activity during and after cancer treatment: an American Cancer Society guide for informed choices [29]	Not specified	Maximise intake	Limit fat in cases of regurgitation	Not specified	Strong flavours and acidic foods (if poorly tolerated)	As required
	2006	Nutrition and physical activity during and after cancer treatment: an American Cancer Society guide for informed choices [30]	Not specified	Maximise intake	Limit fat in cases of regurgitation	Not specified	Strong flavours and acidic foods (if poorly tolerated)	As required
	2016	American Cancer Society Head and Neck Cancer Survivorship Care Guideline [33]	Achieve and maintain a healthy weight	Not specified	Limit saturated fats	High intake	Alcohol, spicy/abrasive foods, extreme-temperature liquids, sugar-containing gum, sugary drinks, acidic foods and drinks	Not specified
	2022	American Cancer Society nutrition and physical activity guideline for cancer survivors [34]	Evidence unclear	Not specified	Not specified	High intake	Alcohol	Not specified
European Head and Neck Society	2022	European Head and Neck Society recommendations for head and neck cancer survivorship care [45]	Achieve and maintain a healthy weight	Consider “nutrition support”	Limit saturated fats	High intake	Alcohol, spicy/abrasive foods, extreme-temperature liquids, sugar-containing gum, sugary drinks, acidic foods and drinks	As required

## Data Availability

The original contributions presented in this study are included in the article/Supplementary Materials. Further inquiries can be directed to the corresponding author.

## Supplementary Materials

The following supporting information can be downloaded at: https://www.mdpi.com/article/10.3390/nu18101516/s1, Table S1: AGREE II instrument scaled and raw domain scores; Table S2: AGREE-REX instrument scaled and raw item means; Figure S1: AGREE II instrument scaled item means; Table S3: AGREE II and AGREE-REX raw data.

## Abbreviations

The following abbreviations are used in this manuscript:

AGREE II	Appraisal of Guidelines for Research & Evaluation, version II
AGREE-REX	Appraisal of Guidelines for Research and Evaluation: Recommendation Excellence
PRISMA	Preferred Reporting Items for Systematic reviews and Meta-Analyses
ASCO	American Society of Clinical Oncology
ESPEN	The European Society for Clinical Nutrition and Metabolism
SEOM	Sociedad Española de Oncología Médica (Spanish Society of Medical Oncology)
NCCN	National Comprehensive Cancer Network
WCRF	World Cancer Research Fund
AICR	American Institute for Cancer Research
SNFGE	Société Nationale Française de Gastroentérologie
FFCD	Fédération Francophone de Cancérologie Digestive
GERCOR	Groupe Coopérateur multidisciplinaire en Oncologie
UNI-CANCER	Fédération Nationale des Centres de Lutte Contre le Cancer
SFCD	Société Française de Chirurgie Digestive
SFED	Société Française d’Endoscopie Digestive
SFRO	Société Française de Radiothérapie Oncologique
ACHBT	Association de Chirurgie Hépato-Bilio-Pancréatique et Transplantation
AFC	Association Française de Chirurgie
SFP-APA	Société Française des Professionnels en Activité Physique Adaptée
SFNCM	Société Francophone de Nutrition Clinique et Métabolisme
AFSOS	Association Francophone pour Soins Oncologiques de Support

## References

[1] Panteli D., Legido-Quigley H., Reichebner C., Ollenschläger G., Schäfer C., Busse R. (2019). Clinical Practice Guidelines as a quality strategy. Improving Healthcare Quality in Europe: Characteristics, Effectiveness and Implementation of Different Strategies.

[2] Guerra-Farfan E., Garcia-Sanchez Y., Jornet-Gibert M., Nuñez J.H., Balaguer-Castro M., Madden K. (2023). Clinical practice guidelines: The good, the bad, and the ugly. Injury.

[3] Yang G.X., Dou S.Q., Liu X.B., Que T., Tang Y., Wang X., Yan L., Zhou L., Jin C., Wang Y. (2025). Quality problems in clinical practice guidelines and guideline appraisal studies: Should we tolerate or eradicate?. J. Eval. Clin. Pract..

[4] Force L.M., Kocarnik J.M., May M.L., Bhangdia K., Crist A., Penberthy L., Pritchett N., Acheson A., Deitesfeld L. (2025). The global, regional, and national burden of cancer, 1990–2023, with forecasts to 2050: A systematic analysis for the Global Burden of Disease Study 2023. Lancet.

[5] Zafar A., Khatoon S., Khan M.J., Abu J., Naeem A. (2025). Advancements and limitations in traditional anti-cancer therapies: A comprehensive review of surgery, chemotherapy, radiation therapy, and hormonal therapy. Discov. Oncol..

[6] Bray F., Laversanne M., Sung H., Ferlay J., Siegel R.L., Soerjomataram I., Jemal A. (2024). Global cancer statistics 2022: GLOBOCAN estimates of incidence and mortality worldwide for 36 cancers in 185 countries. CA Cancer J. Clin..

[7] Global Cancer Burden Growing, Amidst Mounting Need for Services 2024. https://www.who.int/news/item/01-02-2024-global-cancer-burden-growing--amidst-mounting-need-for-services.

[8] Australian Institute of Health and Welfare, Australian Government (2025). Cancer Data in Australia. https://www.aihw.gov.au/reports/cancer/cancer-data-in-australia/contents/about.

[9] Gegechkori N., Haines L., Lin J.J. (2017). Long-Term and Latent Side Effects of Specific Cancer Types. Med. Clin. N. Am..

[10] Stein K.D., Syrjala K.L., Andrykowski M.A. (2008). Physical and psychological long-term and late effects of cancer. Cancer.

[11] Salas S., Cottet V., Dossus L., Fassier P., Ginhac J., Latino-Martel P., Romieu I., Schneider S., Srour B., Touillaud M. (2022). Nutritional Factors during and after Cancer: Impacts on Survival and Quality of Life. Nutrients.

[12] Jiang C., Deng L., Karr M.A., Wen Y., Wang Q., Perimbeti S., Shapiro C.L., Han X. (2022). Chronic comorbid conditions among adult cancer survivors in the United States: Results from the National Health Interview Survey, 2002–2018. Cancer.

[13] Brown J., Byers T., Thompson K., Eldridge B., Doyle C., Williams A.M. (2001). Nutrition During and After Cancer Treatment: A Guide for Informed Choices by Cancer Survivors. CA Cancer J. Clin..

[14] Li Z., Ding X., Chen Y., Keaver L., Champ C.E., Fink C.L., Lebovits S.C., Corroto M., Zhang F.F. (2024). Review of Nutrition Guidelines and Evidence on Diet and Survival Outcomes for Cancer Survivors: Call for Integrating Nutrition into Oncology Care. J. Nutr..

[15] Dos-Santos-Silva I., Gupta S., Orem J., Shulman L.N. (2022). Global disparities in access to cancer care. Commun. Med..

[16] Lowder S.K., Laderchi C.R., Cerutti N., Parsons K. (2025). Food System Policies: A global snapshot from the Food System Policy Database. Glob. Food Secur..

[17] Cámara M., Giner R.M., González-Fandos E., López-García E., Mañes J., Portillo M.P., Rafecas M., Domínguez L., Martínez J.A. (2021). Food-Based Dietary Guidelines around the World: A Comparative Analysis to Update AESAN Scientific Committee Dietary Recommendations. Nutrients.

[18] Monterrosa E.C., Frongillo E.A., Drewnowski A., de Pee S., Vandevijvere S. (2020). Sociocultural Influences on Food Choices and Implications for Sustainable Healthy Diets. Food Nutr. Bull..

[19] Zhang Y.Y., Chen B.X., Wan Q. (2025). Global, regional, and national burden of nutritional deficiencies spanning from 1990 to 2021, with a focus on the impacts observed during the COVID-19 pandemic. Front. Nutr..

[20] Brouwers M.C., Kho M.E., Browman G.P., Burgers J.S., Cluzeau F., Feder G., Fervers B., Graham I.D., Grimshaw J., Hanna S.E. (2010). AGREE II: Advancing guideline development, reporting and evaluation in health care. CMAJ.

[21] Brouwers M.C., Spithoff K., Kerkvliet K., Alonso-Coello P., Burgers J., Cluzeau F., Férvers B., Graham I., Grimshaw J., Hanna S. (2020). Development and Validation of a Tool to Assess the Quality of Clinical Practice Guideline Recommendations. JAMA Netw. Open.

[22] Noyahr J.K., Tatucu-Babet O.A., Chapple L.S., Barlow C.J., Chapman M.J., Deane A.M., Fetterplace K., Hodgson C.L., Winderlich J., Udy A.A. (2022). Methodological Rigor and Transparency in Clinical Practice Guidelines for Nutrition Care in Critically Ill Adults: A Systematic Review Using the AGREE II and AGREE-REX Tools. Nutrients.

[23] Santero M., de Mas J., Rifà B., Clavero I., Rexach I., Bonfill Cosp X. (2024). Assessing the methodological strengths and limitations of the Spanish Society of Medical Oncology (SEOM) guidelines: A critical appraisal using AGREE II and AGREE-REX tool. Clin. Transl. Oncol..

[24] Covidence Systematic Review Software, Veritas Health Innovation, Melbourne, Australia. http://www.covidence.org.

[25] Page M.J., McKenzie J.E., Bossuyt P.M., Boutron I., Hoffmann T.C., Mulrow C.D., Shamseer L., Tetzlaff J.M., Akl E.A., Brennan S.E. (2021). The PRISMA 2020 statement: An updated guideline for reporting systematic reviews. BMJ.

[26] Neuzillet C., Anota A., Foucaut A.M., Védie A.L., Antoun S., Barnoud D., Bouleuc C., Chorin F., Cottet V., Fontaine E. (2021). Nutrition and physical activity: French intergroup clinical practice guidelines for diagnosis, treatment and follow-up (SNFGE, FFCD, GERCOR, UNICANCER, SFCD, SFED, SFRO, ACHBT, AFC, SFP-APA, SFNCM, AFSOS). BMJ Support. Palliat. Care.

[27] Runowicz C.D., Leach C.R., Henry N.L., Henry K.S., Mackey H.T., Cowens-Alvarado R.L., Cannady R.S., Pratt-Chapman M.L., Edge S.B., Jacobs L.A. (2016). American Cancer Society/American Society of Clinical Oncology Breast Cancer Survivorship Care Guideline. CA Cancer J. Clin..

[28] Clinton S.K., Giovannucci E.L., Hursting S.D. (2020). The World Cancer Research Fund/American Institute for Cancer Research Third Expert Report on Diet, Nutrition, Physical Activity, and Cancer: Impact and Future Directions. J. Nutr..

[29] Brown J.K., Byers T., Doyle C., Coumeya K.S., Demark-Wahnefried W., Kushi L.H., McTieman A., Rock C.L., Aziz N., Bloch A.S. (2003). Nutrition and physical activity during and after cancer treatment: An American Cancer Society guide for informed choices. CA Cancer J. Clin..

[30] Doyle C., Kushi L.H., Byers T., Courneya K.S., Demark-Wahnefried W., Grant B., McTiernan A., Rock C.L., Thompson C., Gansler T. (2006). Nutrition and physical activity during and after cancer treatment: An American Cancer Society guide for informed choices. CA Cancer J. Clin..

[31] Skolarus T.A., Wolf A.M., Erb N.L., Brooks D.D., Rivers B.M., Underwood W., Salner A.L., Zelefsky M.J., Aragon-Ching J.B., Slovin S.F. (2014). American Cancer Society prostate cancer survivorship care guidelines. CA Cancer J. Clin..

[32] El-Shami K., Oeffinger K.C., Erb N.L., Willis A., Bretsch J.K., Pratt-Chapman M.L., Cannady R.S., Wong S.L., Rose J., Barbour A.L. (2015). American Cancer Society Colorectal Cancer Survivorship Care Guidelines. CA Cancer J. Clin..

[33] Cohen E.E., LaMonte S.J., Erb N.L., Beckman K.L., Sadeghi N., Hutcheson K.A., Stubblefield M.D., Abbott D.M., Fisher P.S., Stein K.D. (2016). American Cancer Society Head and Neck Cancer Survivorship Care Guideline. CA Cancer J. Clin..

[34] Rock C.L., Thomson C.A., Sullivan K.R., Howe C.L., Kushi L.H., Caan B.J., Neuhouser M.L., Bandera E.V., Wang Y., Robien K. (2022). American Cancer Society nutrition and physical activity guideline for cancer survivors. CA Cancer J. Clin..

[35] American Cancer Society (2022). Nutrition, Physical Activity, Body Weight, and Cancer Survivorship. https://acs4ccc.org/the-new-nutrition-physical-activity-body-weight-and-cancer-survivorship-series/.

[36] Ligibel J.A., Alfano C.M., Courneya K.S., Demark-Wahnefried W., Burger R.A., Chlebowski R.T., Fabian C.J., Gucalp A., Hershman D.L., Hudson M.M. (2014). American Society of Clinical Oncology position statement on obesity and cancer. J. Clin. Oncol..

[37] Shapiro C.L., Poznak C.V., Lacchetti C., Kirshner J., Eastell R., Gagel R., Smith S., Edwards B.J., Frank E., Lyman G.H. (2019). Management of Osteoporosis in Survivors of Adult Cancers With Nonmetastatic Disease: ASCO Clinical Practice Guideline. J. Clin. Oncol..

[38] Arends J., Bachmann P., Baracos V., Barthelemy N., Bertz H., Bozzetti F., Fearon K., Hütterer E., Isenring E., Kaasa S. (2017). ESPEN guidelines on nutrition in cancer patients. Clin. Nutr..

[39] Muscaritoli M., Arends J., Bachmann P., Baracos V., Barthelemy N., Bertz H., Bozzetti F., Hütterer E., Isenring E.A., Kaasa S. (2021). ESPEN practical guideline: Clinical Nutrition in cancer. Clin. Nutr..

[40] Denlinger C.S., Ligibel J.A., Are M., Baker K.S., Demark-Wahnefried W., Dizon D., Friedman D.L., Goldman M., Jones L., King A. (2014). Survivorship: Nutrition and weight management, Version 2.2014. Clinical practice guidelines in oncology. J. Natl. Compr. Cancer Netw..

[41] Sanft T., Day A., Peterson L., Rodriguez M.A., Ansbaugh S., Armenian S., Baker K.S., Ballinger T., Broderick G., Demark-Wahnefried W. (2022). NCCN Guidelines^®^ Insights: Survivorship, Version 1.2022. J. Natl. Compr. Cancer Netw..

[42] Sanft T., Day A.T., Ansbaugh S.M., Ariza-Heredia E.J., Armenian S., Baker K.S., Ballinger T.J., Cathcart-Rake E.J., Cohen S.H., Evgeniou E. (2025). NCCN Guidelines^®^ Insights: Survivorship, Version 2.2025. J. Natl. Compr. Cancer Netw..

[43] Krebs J., Weijenberg M., Baskin M., Lewis S., Copson E., Seidell J., Chan D.S.M., Abar L., Cariolou M., Nanu N. (2024). Diet, Nutrition, Physical Activity and Body Weight for People Living with and Beyond Breast Cancer.

[44] Poling P.E. (2023). Care of Cancer Survivors: Nutrition and Physical Activity. FP Essent..

[45] Verdonck-de Leeuw I., Dawson C., Licitra L., Eriksen J.G., Hosal S., Singer S., Laverty D.P., Golusinski W., Machczynski P., Gomes A.V. (2022). European Head and Neck Society recommendations for head and neck cancer survivorship care. Oral Oncol..

[46] de Las Peñas R., Majem M., Perez-Altozano J., Virizuela J.A., Cancer E., Diz P., Donnay O., Hurtado A., Jimenez-Fonseca P., Ocon M.J. (2019). SEOM clinical guidelines on nutrition in cancer patients (2018). Clin. Transl. Oncol..

[47] Petrakis N.L., Barnes S., King E.B., Lowenstein J., Wiencke J., Lee M.M., Miike R., Kirk M., Coward L. (1996). Stimulatory influence of soy protein isolate on breast secretion in pre- and postmenopausal women. Cancer Epidemiol. Biomark. Prev..

[48] Boutas I., Kontogeorgi A., Dimitrakakis C., Kalantaridou S.N. (2022). Soy Isoflavones and Breast Cancer Risk: A Meta-analysis. In Vivo.

[49] Guha N., Kwan M.L., Quesenberry C.P., Weltzien E.K., Castillo A.L., Caan B.J. (2009). Soy isoflavones and risk of cancer recurrence in a cohort of breast cancer survivors: The Life After Cancer Epidemiology study. Breast Cancer Res. Treat..

[50] Bakaloudi D.R., Halloran A., Rippin H.L., Oikonomidou A.C., Dardavesis T.I., Williams J., Wickramasinghe K., Breda J., Chourdakis M. (2021). Intake and adequacy of the vegan diet. A systematic review of the evidence. Clin. Nutr..

[51] Russell C., Whelan J., Love P. (2022). Assessing the Cost of Healthy and Unhealthy Diets: A Systematic Review of Methods. Curr. Nutr. Rep..

[52] Escoto K.H., Laska M.N., Larson N., Neumark-Sztainer D., Hannan P.J. (2012). Work hours and perceived time barriers to healthful eating among young adults. Am. J. Health Behav..

[53] Baird S., Moran R., Hacker S., Lawton D., Hill L. (2025). Examining the cost burden of dietary supplements in older adults: An analysis from the AAA longroad study. BMC Geriatr..

[54] Martínez García L., Sanabria A.J., García Alvarez E., Trujillo-Martín M.M., Etxeandia-Ikobaltzeta I., Kotzeva A., Rigau D., Louro-González A., Barajas-Nava L., del Campo P.D. (2014). The validity of recommendations from clinical guidelines: A survival analysis. CMAJ.

[55] Zhou P., Chen L., Wu Z., Wang E., Yan Y., Guan X., Zhai S., Yang K. (2023). The barriers and facilitators for the implementation of clinical practice guidelines in healthcare: An umbrella review of qualitative and quantitative literature. J. Clin. Epidemiol..

[56] Magwood O., Riddle A., Petkovic J., Lytvyn L., Khabsa J., Atwere P., Akl E.A., Campbell P., Welch V., Smith M. (2022). PROTOCOL: Barriers and facilitators to stakeholder engagement in health guideline development: A qualitative evidence synthesis. Campbell Syst. Rev..

[57] Bryant E.A., Thomas R., Sanders S.L. (2026). Patient and Public Involvement Reporting Consistency: Analysis of 24 Clinical Practice Guidelines From 4 Countries. Clin. Public Health Guidel..

[58] Graham R., Mancher M., Miller Wolman D., Greenfield S., Steinberg E. (2011). Institute of Medicine Committee on Standards for Developing Trustworthy Clinical Practice G. Clinical Practice Guidelines We Can Trust.

[59] Bryant E.A., Scott A.M., Greenwood H., Thomas R. (2022). Patient and public involvement in the development of clinical practice guidelines: A scoping review. BMJ Open.

[60] Zhou H.J., Deng L.J., Wang T., Chen J.X., Jiang S.Z., Yang L., Liu F., Weng M.-H., Hu J.-W., Tan J.-Y. (2021). Clinical practice guidelines for the nutritional risk screening and assessment of cancer patients: A systematic quality appraisal using the AGREE II instrument. Support. Care Cancer.

[61] Fuentes Padilla P., Martínez G., Vernooij R.W.M., Cosp X.B., Alonso-Coello P. (2016). Nutrition in critically ill adults: A systematic quality assessment of clinical practice guidelines. Clin. Nutr..

[62] Bauer M.S. (2002). A review of quantitative studies of adherence to mental health clinical practice guidelines. Harv. Rev. Psychiatry.

[63] Bierbaum M., Rapport F., Arnolda G., Tran Y., Nic Giolla Easpaig B., Ludlow K., Clay-Williams R., Austin E., Laginha B., Lo C.Y. (2023). Rates of adherence to cancer treatment guidelines in Australia and the factors associated with adherence: A systematic review. Asia Pac. J. Clin. Oncol..

[64] Shehu E., Kugler C.M., Schäfer N., Rosen D., Schaefer C., Kötter T., Follmann M., Pieper D. (2025). Barriers and facilitators of adherence to clinical practice guidelines in Germany-A systematic review. J. Eval. Clin. Pract..

[65] Qumseya B., Goddard A., Qumseya A., Estores D., Draganov P.V., Forsmark C. (2021). Barriers to Clinical Practice Guideline Implementation Among Physicians: A Physician Survey. Int. J. Gen. Med..

[66] Wang T., Tan J.-Y., Liu X.-L., Zhao I. (2023). Barriers and enablers to implementing clinical practice guidelines in primary care: An overview of systematic reviews. BMJ Open.

[67] Covitt B.A., Anderson C.W. (2022). Untangling Trustworthiness and Uncertainty in Science: Implications for Science Education. Sci. Educ..

[68] Baughman C., Norman K., Mukamal K. (2024). Adherence to American Cancer Society Nutrition and Physical Activity Guidelines Among Cancer Survivors. JAMA Oncol..

[69] Park S.H., Knobf M.T., Kerstetter J., Jeon S. (2019). Adherence to American Cancer Society Guidelines on Nutrition and Physical Activity in Female Cancer Survivors: Results From a Randomized Controlled Trial (Yale Fitness Intervention Trial). Cancer Nurs..

[70] Wang Y., Newton C.C., McCullough M.L., Teras L.R., Bodelon C., Rees-Punia E., Um C.Y., Makaroff L., Patel A.V. (2025). Following the American Cancer Society guideline for cancer survivors and obesity-related cancer survival. J. Natl. Cancer Inst..

[71] Greenberg A.L., Tolstykh I.V., Van Loon K., Laffan A., Stanfield D., Steiding P., Kenfield S.A., Chan J.M., Atreya C.E., Piawah S. (2023). Association between adherence to the American Cancer Society Nutrition and Physical Activity Guidelines and stool frequency among colon cancer survivors: A cohort study. J. Cancer Surviv..

[72] Kang M., Song S., Cho H.J., Kim Z., Youn H.J., Cho J., Min J.W., Kim Y.S., Choi S.-W., Lee J.E. (2024). Adherence to the American Cancer Society guidelines on nutrition and physical activity for cancer survivors and biomarkers of inflammation among breast cancer survivors. Epidemiol. Health.

[73] Bozzetti F., Cotogni P. (2020). Nutritional Issues in Head and Neck Cancer Patients. Healthcare.

[74] Molnár A., Pálfi E., Belák B., Blasszauer C., Reibl D., Lövey J. (2024). Positive correlation between persistence of medical nutrition therapy and overall survival in patients with head and neck cancer. Pathol. Oncol. Res..

[75] Kabat G.C., Matthews C.E., Kamensky V., Hollenbeck A.R., Rohan T.E. (2015). Adherence to cancer prevention guidelines and cancer incidence, cancer mortality, and total mortality: A prospective cohort study. Am. J. Clin. Nutr..

[76] Cash A.H., Hamre B.K., Pianta R.C., Myers S.S. (2012). Rater calibration when observational assessment occurs at large scale: Degree of calibration and characteristics of raters associated with calibration. Early Child. Res. Q..

