# Supplementary material for: Nutrition Recommendations for People Living After Cancer: A Systematic Review and Critical Appraisal of Clinical Practice Guidelines
Source: Nutrients. 2026 May 9;18(10):1516. doi: 10.3390/nu18101516 (PMC13210353; doi:10.3390/nu18101516)
Supplement: Supplementary file 1 [file nutrients-18-01516-s001.zip › Supplementary tables and figures.pdf]

Supplementary Table S1. AGREE II Instrument Scaled and Raw Domain Scores

Organisation	Year	Title of CPG	Domain 1 scaled* (raw)	Domain 2 scaled* (raw)	Domain 3 scaled* (raw)	Domain 4 scaled* (raw)	Domain 5 scaled* (raw)	Domain 6 scaled* (raw)	Total scaled* (raw)
American Academy of Family Physicians	2023	Care of Cancer Survivors: Nutrition and Physical Activity	42% (21)	25% (15)	29% (44)	58% (27)	19% (17)	71% (21)	36% (145)
American Cancer Society (ACS)	2001	Nutrition during and after cancer treatment: a guide for informed choices by cancer survivors	61% (28)	53% (25)	25% (40)	75% (33)	15% (15)	71% (21)	42% (162)
	2003	Nutrition and physical activity during and after cancer treatment: an American Cancer Society guide for informed choices	81% (35)	67% (30)	30% (45)	81% (35)	6% (11)	71% (21)	47% (177)
	2006	Nutrition and physical activity during and after cancer treatment: an American Cancer Society guide for informed choices	86% (37)	58% (27)	44% (58)	72% (32)	13% (14)	71% (21)	52% (189)
	2014	American Cancer Society Prostate Cancer Survivorship Care Guidelines	56% (26)	56% (26)	65% (78)	75% (33)	23% (19)	100% (28)	59% (210)
	2015	American Cancer Society Colorectal Cancer Survivorship Care Guidelines	92% (39)	64% (29)	75% (88)	89% (38)	38% (26)	100% (28)	73% (248)
	2016	American Cancer Society Head and Neck Cancer Survivorship Care Guideline	89% (38)	67% (30)	75% (88)	75% (33)	23% (19)	100% (28)	69% (236)
	2022	American Cancer Society nutrition and physical activity guideline for cancer survivors	97% (41)	78% (34)	77% (90)	92% (39)	65% (39)	100% (28)	82% (271)
	2022	Nutrition, Physical Activity, Body Weight and Cancer Survivorship	89% (38)	72% (32)	56% (70)	92% (39)	75% (44)	75% (22)	72% (245)
American Cancer Society/American Society for Clinical Oncology (ACS/ASCO)	2016	American Cancer Society/American Society for Clinical Oncology Breast Cancer Survivorship Care Guideline	100% (42)	78% (34)	91% (103)	94% (40)	58% (36)	100% (28)	86% (283)
American Society for Clinical Oncology (ASCO)	2015	American Society of Clinical Oncology Position Statement on Obesity and Cancer	47% (23)	33% (18)	18% (33)	58% (27)	23% (19)	100% (28)	37% (148)
	2019	Management of Osteoporosis in Survivors of Adult Cancers With Nonmetastatic Disease: ASCO Clinical Practice Guideline	92% (39)	64% (29)	79% (92)	92% (39)	77% (45)	100% (28)	82% (272)
European Head and Neck Society	2022	European Head and Neck Society recommendations for head and neck cancer survivorship care	100% (42)	61% (28)	70% (83)	89% (38)	50% (32)	96% (27)	74% (250)

Supplementary Table S1. *Cont.*

Organisation	Year	Title of CPG	Domain 1 scaled* (raw)	Domain 2 scaled* (raw)	Domain 3 scaled* (raw)	Domain 4 scaled* (raw)	Domain 5 scaled* (raw)	Domain 6 scaled* (raw)	Total scaled* (raw)
European Society for Clinical Nutrition and Metabolism (ESPEN)	2017	ESPEN guidelines on nutrition in cancer patients	97% (41)	67% (30)	90% (102)	89% (38)	69% (41)	100% (28)	85% (280)
	2021	ESPEN practical guideline: Clinical Nutrition in cancer	83% (36)	81% (35)	60% (74)	81% (35)	46% (30)	75% (22)	67% (232)
European Society for Medical Oncology (SEOM)	2018	SEOM clinical guidelines on nutrition in cancer patients (2018)	61% (28)	31% (17)	47% (61)	78% (34)	27% (21)	46% (15)	47% (176)
National Comprehensive Cancer Network (NCCN)	2014	Survivorship: nutrition and weight management, Version 2.2014. Clinical practice guidelines in oncology	64% (29)	50% (24)	65% (78)	72% (32)	42% (28)	67% (20)	60% (211)
	2022	NCCN Guidelines Insights: Survivorship, Version 1.2022	92% (39)	64% (29)	40% (54)	89% (38)	27% (21)	83% (24)	58% (205)
	2025	NCCN Guidelines Insights: Survivorship, Version 2.2025	83% (36)	61% (28)	48% (62)	92% (39)	50% (32)	71% (21)	62% (218)
SNFGE[a], FFCD[b], GERCOR[c], UNICANCER[d], SFCD[e], SFED[f], SFRO[g], ACHBT[h], AFC[i], SFP-APA[j], SFNCM[k], AFSOS[l]	2021	Nutrition and physical activity: French intergroup clinical practice guidelines for diagnosis, treatment and follow-up (SNFGE, FFCD, GERCOR, UNICANCER, SFCD, SFED, SFRO, ACHBT, AFC, SFP-APA, SFNCM, AFSOS	72% (32)	44% (22)	64% (77)	89% (38)	52% (33)	71% (21)	64% (223)
World Cancer Research Fund (WCRF)	2024	Diet, nutrition, physical activity and body weight for people living with and beyond breast cancer	94% (40)	78% (34)	84% (97)	75% (33)	33% (24)	46% (15)	71% (243)
World Cancer Research Fund/American Institute for Cancer Research (WCRF/AICR)	2020	The World Cancer Research Fund/American Institute for Cancer Research Third Expert Report on Diet, Nutrition, Physical Activity, and Cancer: Impact and Future Directions	61% (28)	39% (20)	58% (72)	61% (28)	33% (24)	63% (19)	53% (191)
		**Mean (range)** (Raw score)	**79% (42-100%)** (34.5)	**59% (25-81%)** (27.1)	**59% (18-91%)** (72.2)	**80% (58-94%)** (34.9)	**39% (6-77%)** (26.8)	**81% (46-100%)** (23.4)	**63% (36-86%)** (218.9)

*Calculated according to the method outlined in the AGREE II manual; [a] Société Nationale Française de Gastroentérologie; [b] Fédération Francophone de Cancérologie Digestive; [c] Groupe Coopérateur multidisciplinaire en Oncologie; [d] Fédération Nationale des Centres de Lutte Contre le Cancer; [e] Société Française de Chirurgie Digestive; [f] Société Française d'Endoscopie Digestive; [g] Société Française de Radiothérapie Oncologique; [h] Association de Chirurgie Hépato-Bilio-Pancréatique et Transplantation; [i] Association Française de Chirurgie; [j] Société Française des Professionnels en Activité Physique Adaptée; [k] Société Francophone de Nutrition Clinique et Métabolisme; [l] Association Francophone pour Soins Oncologiques de Support

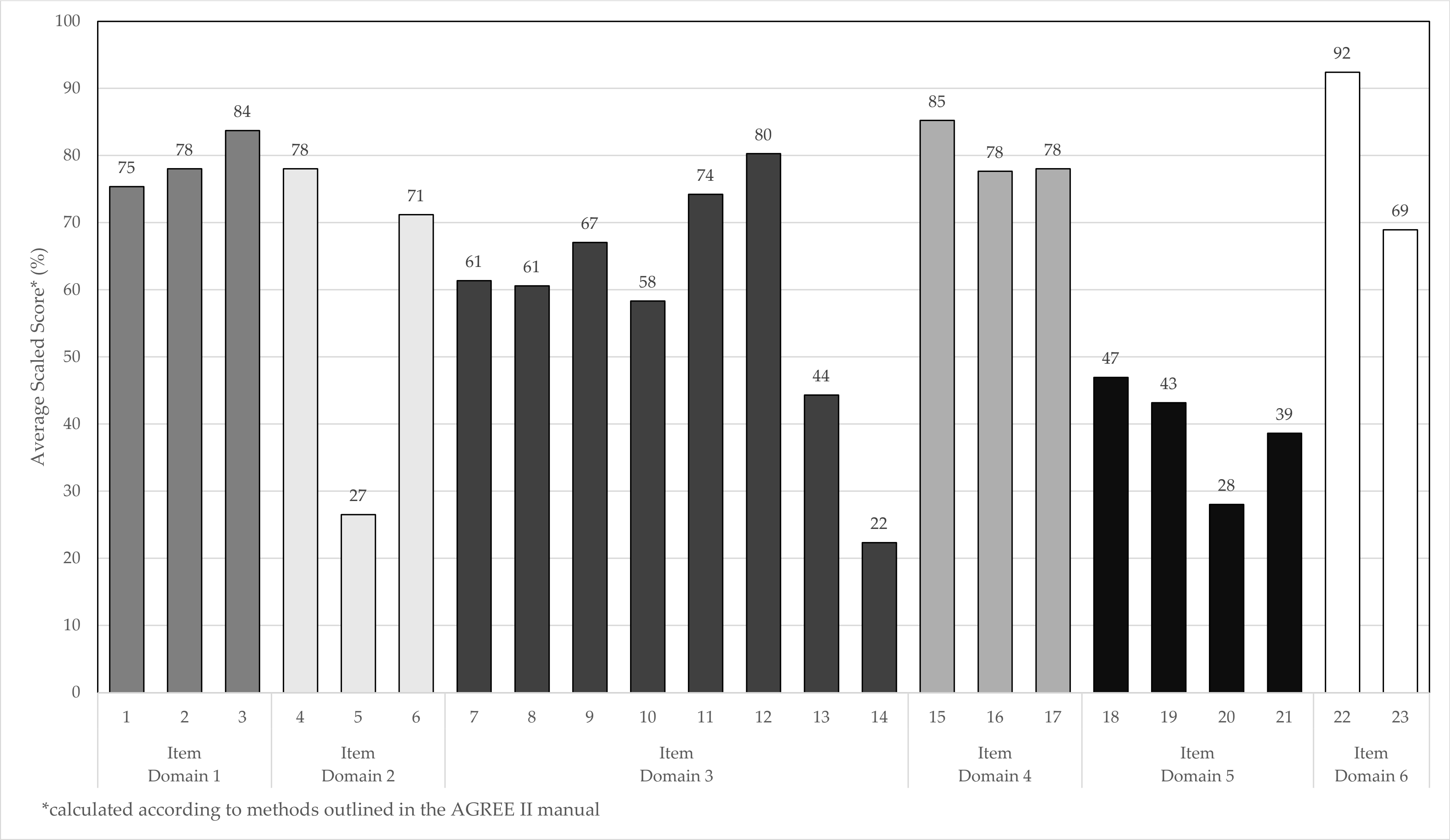

Supplementary Figure S1. AGREE II Instrument Scaled Item Means

Supplementary Table S2. AGREE-REX Instrument Scaled and Raw Item Means

Organisation/ publishing group	Year	Title of CPG	Item 1 scaled* (raw)	Item 2 scaled* (raw)	Item 3 scaled* (raw)	Item 4 scaled* (raw)	Item 5 scaled* (raw)	Item 6 scaled* (raw)	Item 7 scaled* (raw)	Item 8 scaled* (raw)	Item 9 scaled* (raw)	Total scaled* (raw)	Recommendation for use in appropriate context
American Academy of Family Physicians	2023	Care of Cancer Survivors: Nutrition and Physical Activity	42% (7)	50% (8)	42% (7)	8% (3)	17% (4)	8% (3)	8% (3)	25% (5)	17% (4)	24% (44)	No
American Cancer Society (ACS)	2001	Nutrition during and after cancer treatment: a guide for informed choices by cancer survivors	25% (5)	42% (7)	42% (7)	33% (6)	17% (4)	8% (3)	8% (3)	25% (5)	8% (3)	23% (43)	No
	2003	Nutrition and physical activity during and after cancer treatment: an American Cancer Society guide for informed choices	58% (9)	50% (8)	42% (7)	42% (7)	17% (4)	8% (3)	8% (3)	33% (6)	8% (3)	30% (50)	Yes, with modifications
	2006	Nutrition and physical activity during and after cancer treatment: an American Cancer Society guide for informed choices	58% (9)	42% (7)	33% (6)	17% (4)	25% (5)	8% (3)	8% (3)	33% (6)	17% (4)	27% (47)	No
	2014	American Cancer Society Prostate Cancer Survivorship Care Guidelines	67% (10)	83% (12)	33% (6)	25% (5)	17% (4)	17% (4)	25% (5)	75% (11)	25% (5)	41% (62)	Yes, with modifications
	2015	American Cancer Society Colorectal Cancer Survivorship Care Guidelines	83% (12)	83% (12)	50% (8)	42% (7)	17% (4)	8% (3)	67% (10)	67% (10)	25% (5)	49% (71)	Yes
	2016	American Cancer Society Head and Neck Cancer Survivorship Care Guideline	83% (12)	83% (12)	50% (8)	50% (8)	25% (5)	8% (3)	67% (10)	58% (9)	25% (5)	50% (72)	Yes
	2022	American Cancer Society nutrition and physical activity guideline for cancer survivors	92% (13)	67% (10)	50% (8)	25% (5)	33% (6)	25% (5)	75% (11)	75% (11)	50% (8)	55% (77)	Yes
	2022	Nutrition, Physical Activity, Body Weight and Cancer Survivorship	67% (10)	83% (12)	67% (10)	67% (10)	33% (6)	17% (4)	17% (4)	75% (11)	50% (8)	53% (75)	Yes
American Cancer Society/American Society for Clinical Oncology (ACS/ASCO)	2016	American Cancer Society/American Society for Clinical Oncology Breast Cancer Survivorship Care Guideline	100% (14)	92% (13)	83% (12)	83% (12)	75% (11)	42% (7)	75% (11)	100% (14)	50% (8)	78% (102)	Yes

Supplementary Table S2. *Cont.*

Organisation/ publishing group	Year	Title of CPG	Item 1 scaled* (raw)	Item 2 scaled* (raw)	Item 3 scaled* (raw)	Item 4 scaled* (raw)	Item 5 scaled* (raw)	Item 6 scaled* (raw)	Item 7 scaled* (raw)	Item 8 scaled* (raw)	Item 9 scaled* (raw)	Total scaled* (raw)	Recommendation for use in appropriate context
American Society for Clinical Oncology (ASCO)	2015	American Society of Clinical Oncology Position Statement on Obesity and Cancer	42% (7)	33% (6)	33% (6)	0% (2)	0% (2)	42% (7)	50% (8)	50% (8)	42% (7)	32% (53)	No
	2019	Management of Osteoporosis in Survivors of Adult Cancers With Nonmetastatic Disease: ASCO Clinical Practice Guideline	92% (13)	58% (9)	67% (10)	67% (10)	58% (9)	75% (11)	25% (5)	92% (13)	25% (5)	62% (85)	Yes, with modifications
European Head and Neck Society	2022	European Head and Neck Society recommendations for head and neck cancer survivorship care	92% (13)	75% (11)	50% (8)	8% (3)	8% (3)	25% (5)	33% (6)	83% (12)	33% (6)	45% (67)	Yes, with modifications
European Society for Clinical Nutrition and Metabolism (ESPEN)	2017	ESPEN guidelines on nutrition in cancer patients	100% (14)	100% (14)	75% (11)	50% (8)	8% (3)	67% (10)	75% (11)	83% (12)	50% (8)	68% (91)	Yes
	2021	ESPEN practical guideline: Clinical Nutrition in cancer	58% (9)	75% (11)	50% (8)	75% (11)	33% (6)	42% (7)	42% (7)	75% (11)	33% (6)	54% (76)	Yes, with modifications
European Society for Medical Oncology (SEOM)	2018	SEOM clinical guidelines on nutrition in cancer patients (2018)	67% (10)	67% (10)	42% (7)	42% (7)	17% (4)	0% (2)	8% (3)	25% (5)	17% (4)	31% (52)	Yes, with modifications
National Comprehensive Cancer Network (NCCN)	2014	Survivorship: nutrition and weight management, Version 2.2014. Clinical practice guidelines in oncology	67% (10)	50% (8)	50% (8)	33% (6)	25% (5)	8% (3)	33% (6)	67% (10)	42% (7)	42% (63)	Yes
	2022	NCCN Guidelines Insights: Survivorship, Version 1.2022	67% (10)	83% (12)	75% (11)	33% (6)	25% (5)	8% (3)	42% (7)	83% (12)	50% (8)	52% (74)	Yes
	2025	NCCN Guidelines Insights: Survivorship, Version 2.2025	83% (12)	75% (11)	75% (11)	33% (6)	33% (6)	8% (3)	25% (5)	100% (14)	67% (10)	56% (78)	Yes
SNFGE[a], FFCD[b], GERCOR[c], UNICANCER[d], SFCD[e], SFED[f], SFRO[g], ACHBT[h], AFC[i], SFP-APA[j], SFNCM[k], AFSOS[l]	2021	Nutrition and physical activity: French intergroup clinical practice guidelines for diagnosis, treatment and follow-up (SNFGE, FFCD, GERCOR, UNICANCER, SFCD, SFED, SFRO, ACHBT, AFC, SFP-APA, SFNCM, AFSOS	75% (11)	67% (10)	42% (7)	58% (9)	17% (4)	0% (2)	58% (9)	75% (11)	42% (7)	48% (70)	Yes, with modifications

Supplementary Table S2. *Cont.*

Organisation/ publishing group	Year	Title of CPG	Item 1 scaled* (raw)	Item 2 scaled* (raw)	Item 3 scaled* (raw)	Item 4 scaled* (raw)	Item 5 scaled* (raw)	Item 6 scaled* (raw)	Item 7 scaled* (raw)	Item 8 scaled* (raw)	Item 9 scaled* (raw)	Total scaled* (raw)	Recommendation for use in appropriate context
World Cancer Research Fund (WCRF)	2024	Diet, nutrition, physical activity and body weight for people living with and beyond breast cancer	92% (13)	75% (11)	67% (10)	67% (10)	58% (9)	42% (7)	33% (6)	92% (13)	58% (9)	65% (88)	Yes
World Cancer Research Fund, American Institute for Cancer Research (WCRF/AICR)	2020	The World Cancer Research Fund/American Institute for Cancer Research Third Expert Report on Diet, Nutrition, Physical Activity, and Cancer: Impact and Future Directions	67% (10)	67% (10)	58% (9)	42% (7)	0% (2)	83% (12)	33% (6)	75% (11)	33% (6)	51% (73)	Yes, with modifications
		**Mean (range)** (raw score)	**72% (25-100%)** (10.6)	**68% (33-100%)** (10.2)	**53% (33-83%)** (8.4)	**41% (0-83%)** (6.9)	**25% (0-75%)** (5.0)	**25% (0-83%)** (5.0)	**37% (8-75%)** (6.5)	**67% (25-100%)** (10.0)	**35% (8-67%)** (6.2)	**47% (23-78%)** (68.8)	

* Calculated according to the method outlined in the AGREE II manual; [a] Société Nationale Française de Gastroentérologie; [b] Fédération Francophone de Cancérologie Digestive; [c] Groupe Coopérateur multidisciplinaire en Oncologie; [d] Fédération Nationale des Centres de Lutte Contre le Cancer; [e] Société Française de Chirurgie Digestive; [f] Société Française d'Endoscopie Digestive; [g] Société Française de Radiothérapie Oncologique; [h] Association de Chirurgie Hépato-Bilio-Pancréatique et Transplantation; [i] Association Française de Chirurgie; [j] Société Française des Professionnels en Activité Physique Adaptée; [k] Société Francophone de Nutrition Clinique et Métabolisme; [l] Association Francophone pour Soins Oncologiques de Support
